# Improving the Measurement of Iron(III) Bioavailability in Freshwater Samples: Methods and Performance

**DOI:** 10.1002/etc.5530

**Published:** 2022-12-20

**Authors:** Emiliano Balsamo Crespo, Amanda Reichelt‐Brushett, Ross E. W. Smith, Andrew L. Rose, Graeme E. Batley

**Affiliations:** ^1^ Faculty of Science and Engineering Southern Cross University East Lismore New South Wales Australia; ^2^ Hydrobiology Milton Queensland Australia; ^3^ CSIRO Land and Water Kirrawee New South Wales Australia

**Keywords:** Bioavailability, water quality guidelines, trace metals, analytical chemistry

## Abstract

The toxicity of iron(III) in fresh waters has been detected at concentrations above the iron solubility limit, indicating a contribution of colloidal and particulate forms of iron(III) to the toxicity response. Current water quality guideline values for iron in fresh water are based on analytical determinations of filterable or total iron. Filtration, however, can underestimate bioavailable iron by retaining some of the colloidal fraction, and total determinations overestimate bioavailable iron measurements by recovering fractions of low bioavailability from suspended solids (e.g., iron oxides and oxyhydroxides) naturally abundant in many surface waters. Consequently, there is a need for an analytical method that permits the determination of a bioavailable iron fraction, while avoiding false‐negative and false‐positive results. Ideally, a measurement technique is required that can be readily applied by commercial laboratories and field sampling personnel, and integrated into established regulatory schemes. The present study investigated the performance of pH 2 and pH 4 extractions to estimate a bioavailable iron(III) fraction in synthetic water samples containing iron phases of different reactivities. The effects of aging on fresh precipitates were also studied. The total recoverable, 0.45‐µm filtered, and pH 4 extractable fractions did not discriminate iron phases and age groups satisfactorily. Contrastingly, the pH 2 extraction showed specificity toward iron phases and aging (0.5–2‐h interval). Extraction times above 4 h and up to 16 h equally recovered >90% of the spiked iron regardless of its age. Furthermore, <1% of the well‐mineralized iron was targeted. The present study shows that a pH 2 dilute‐acid extraction is a suitable candidate method to operationally define iron fractions of higher bioavailability avoiding false‐negative and false‐positive results. *Environ Toxicol Chem* 2023;42:303–316. © 2022 The Authors. *Environmental Toxicology and Chemistry* published by Wiley Periodicals LLC on behalf of SETAC.

## INTRODUCTION

The solubility of iron (Fe) in natural waters is controlled by physicochemical parameters such as pH, oxygen partial pressure, temperature, and the effects of these on the hydrolysis and polymerization products, which readily precipitate at circumneutral pH. Moreover, redox species respond differently to these variables. Hydrolysis of soluble iron(II) (Fe^2+^) occurs at pH >8, whereas hydrolysis of soluble iron(III) (Fe^3+^) occurs at pH >3 (Langmuir, 1997). This translates into higher solubilities for iron(II) species than iron(III) species (e.g., Fe(OH)_2_ log *K*_so_ = −15.1, Fe(OH)_3_ log *K*_so_ = −38.6; Langmuir, 1997; Stefánsson, 2007). However, the oxidation of iron(II) by oxygen additionally limits its presence in oxygenated circumneutral waters and, in turn, cycles into the iron(III) colloidal pool in aquatic systems (Langmuir, 1997; Stumm & Morgan, 1996). Thus, in most surface waters, soluble Fe^3+^ is scarce and its hydrolysis products and precipitates are instead the most abundant species (Fox, 1988; Kleja et al., 2012). These precipitates usually have a small particle size (averaging 2–5 nm) and high surface area, which renders them highly reactive (Hassellov & von der Kammer, 2008). Furthermore, these particles experience transformation due to rearrangement of their crystal structure over time (e.g., due to olation, oxolation [formation of hydroxo and oxo bridges, respectively], and dissolution–reprecipitation), and in turn their reactivity decreases as their crystallinity becomes more stable and orderly (Blesa & Matijević, 1989). Ferrihydrite is one of the most common products of freshly precipitated iron(III), but other oxyhydroxides also occur. For example, hematite and goethite are the two most stable phases of iron oxides and oxyhydroxides, and both are prevalent in weathered lateritic soil profiles such as in Australia, India, and Brazil (e.g., Allard et al., 2011). In general terms, goethite is commonly associated with acidic humid conditions, whereas hematite is prevalent in hot and arid regions (Guo & Barnard, 2013). Another common mineral phase is magnetite, which is formed in soils and sediment under anoxic conditions. Its mixed valence composition makes it unstable in oxic environments, which favours the formation of maghemite instead (Guo & Barnard, 2013).

The transport of iron in surface waters plays a major role in its geochemical cycle. The suspended iron loads in rivers reflect the global average iron crustal concentration (Davison & De Vitre, 1992). For instance, >95% of the iron loads of both the Rio Negro and the Amazon River have been reported to be carried by the particulate fraction (defined as >0.2 μm) despite the large difference in suspended solids loads between the two rivers (Allard et al., 2004). Furthermore, it has been reported that approximately 80%–90% of transported iron in rivers is present as mineralized species. However, in some riverine systems iron bound to natural organic matter in the form of colloidal complexes (colloidal Fe–NOM) comprises approximately 60% of the total suspended load, maintaining the concentration of operationally defined dissolved iron above its theoretical solubility limit (Gibbs, 1973; Olivié‐Lauquet et al., 1999).

High levels of iron in the aquatic environment can lead to negative impacts, affecting habitat conditions and directly or indirectly exerting chemical or physical toxicity on organisms and communities (e.g., Kotalik et al., 2019; and references therein). Laboratory ecotoxicological assessments have detected toxicity at concentrations above the solubility limit of iron(III) and therefore it is suspected that precipitates contribute to the observed toxicity response (e.g., Arbildua et al., 2017). However, from a toxicodynamic perspective, it is not clear what fractions and mineral phases are bioavailable and responsible for toxicity to freshwater organisms (Australian and New Zealand Governments and Australian State and Territory Governments [ANZG], 2020). The reactivity and bioavailability of iron oxyhydroxides depends on a wide range of factors, including aggregate structure, crystallinity, particle size, surface area, coprecipitates, and film passivation among many others, all of which are influenced by variables such as pH, ionic strength, temperature, organic matter loads in the immediate environment, and vary with time (Cornell & Schwertmann, 2003).

Several dissolution techniques and procedures have been developed to operationally define reactive or bioavailable fractions of iron. Generally, these extraction procedures attempt to isolate or remove adsorbed and poorly crystalline fractions in the first steps, and more mineralized phases in the final stages of the sequence. Fraction names and properties vary according to the extractants and the number of steps, and some schemes include an oxidizable fraction or two different levels of reductive dissolution (e.g., hydroxylamine hydrochloride vs. dithionite; see Voelz et al., 2019; and references therein.). These techniques have been widely used, particularly in sediment and soil research, but caution must be exercised because selectivity and efficacy vary, and unintended and undesirable removal of some species during certain steps in the process show the importance of verifying results via alternative techniques (Hepburn et al., 2020). This makes them quite difficult to apply in a streamlined manner by commercial laboratories, requiring attentive and skilled technicians. On the other hand, a list of commonly applied standardized methods is available for routine laboratory operations (American Public Health Association [APHA], 2017; US Environmental Protection Agency [USEPA], 1994). These methods commonly distinguish an operationally defined “dissolved” fraction (0.45‐µm pore size filter), a “soluble or extractable” fraction, and a “total” fraction, with an increasingly aggressive level of sample processing, respectively. Unfortunately, the nomenclature for these methods may, to the technician, be inadvertently confusing. As Table 1 shows, different methods with seemingly the same target fraction use procedures of a disparate nature: whereas the acid‐soluble fraction from USEPA (1991) Method 200.1 is extracted with dilute HNO_3_ at room temperature, the acid‐extractable from APHA 3030C requires a heating step and uses HCl. These differences will undoubtedly have an impact on the amount and type of iron phase which is targeted (e.g., chloride is an enhancer of dissolution for iron oxides; Sulzberger et al., 1989). In general terms, the more aggressive the step in the process (i.e., added heat, stronger acid, or acid mix), the less selective the technique and the more iron will be recovered.

**Table 1:** Standard methods for trace metal analysis in waters

Method	Recovered fraction	Extraction mechanism	Heating	Sample preservation/storage
APHA 3030 B	Dissolved	0.45‐µm pore size membrane filter	No	Store at 4 °C Acidification with concentrated HNO_3_ to pH 2 after filtration
USEPA 200.8‐11.1	Dissolved	0.45‐µm pore size membrane filter	No	Acidification with 50% HNO_3_ to 1% v/v acid (pH <2) after filtration
APHA 3030C	Acid extractable	5 ml of 50% v/v HCl in 100 ml of sample and posterior filtration with 0.45‐µm pore size membrane filter	Yes	Acidification with concentrated HNO_3_ to pH <2
USEPA 200.1	Acid soluble	50% v/v HNO_3_ for 16 h pH 1.75 +/0.1 and posterior filtration with 0.45‐µm pore size membrane filter	No	Acidification with 50% HNO_3_ to 1% v/v acid (pH~2) Store at 4°C for up to 3 days
APHA 3030 Fb	Recoverable	Mix of 2 ml of 50% v/v HNO_3_ and 10 ml of 50% v/v HCl	Yes	Acidification with 50% HNO_3_ to 1% v/v acid (pH <2) Filter or settle digests overnight
USEPA 200.8‐11.2	Total recoverable	2–4 ml of 50% v/v HNO_3_ and 1–10 ml of 20% v/v HCl depending on undissolved solids content	Yes	Acidification with 50% HNO_3_ to 1% v/v acid (pH <2 16 h) Filter or settle digests overnight Make fresh dilutions for analysis if necessary

Percentage values express dilution with respect to volume units.

APHA = American Public Health Association; USEPA = US Environmental protection agency.

Another major challenge in assessing the reactivity of iron precipitates is that equilibrium is altered and iron speciation is affected on sampling. Therefore, processing of samples by most methods (e.g., filtration, centrifugation, acidification) will inevitably introduce artifacts (Lead et al., 1997). For example, Canadian water quality guideline values for iron are based on a determination of total iron at a threshold of 1 mg/L and dissolved iron at 0.35 mg/L (Canadian Council of Resources and Environmental Ministers, 1987; Phippen et al., 2008). In iron‐rich landscapes, such thresholds would be hard to meet and likely do not reflect true environmental risk. The suspended particulate material will likely render total determinations noncompliant despite the low reactivity of well‐mineralized iron oxides. Conversely, if the suspended particulate material contained freshly precipitated iron, its tendency to adsorb to other particles and become filter‐removable in circumneutral pH waters will lead to an underestimation of filtered iron (“dissolved”) which otherwise should be accounted as bioavailable. These issues have been discussed previously by Markich et al. (2001), who proposed the inclusion of bioavailability as a criterion to be considered in the assessment of environmental risk and the decision‐making process. Furthermore, in the last revision (draft published for public consultation) of the Australian and New Zealand water quality guidelines for iron in fresh waters, the concern for false‐negative and false‐positive results was highlighted (ANZG, 2020).

Not surprisingly, with the wide range of iron species present in the environment, and the many approaches to define them, no particular extraction technique can encompass the complexity of iron speciation and capture every potential input of bioavailable iron in the aquatic environment. However, appropriate approximations may be achieved by avoiding the extraction of iron species of relatively low reactivity. This would be a priority when it comes to assessing bioavailable iron fractions in turbid water with a high content of suspended particulates. The objective of the present study was to investigate two main aspects regarding the definition of a bioavailable iron fraction. 1) The performance of commercially used methods to extract reactive iron under conditions conducive to false positives or false negatives. 2) The effect of aging on the reactivity of iron species in water samples, and its implications for the logistics of sample transportation and processing.

From an environmental management perspective, there is a need for a method that prevents the inefficient use of resources due to false noncompliant values resulting from the complexity involved in the sampling of iron in natural waters. Thus, the aim of the present study is to provide a body of evidence which will contribute to more efficient water quality management.

## MATERIALS AND METHODS

### Reagents and formulation of synthetic medium

All reagents used for the experiments were analytical grade and all solutions were prepared using 18‐MΩ.cm resistivity Milli‐Q water. All labware and bottles were routinely acid‐washed for at least 48 h in 10% v/v HCl/HNO_3_ and thoroughly rinsed with Milli‐Q water. To avoid cross‐contamination, bottles were labelled for the type of treatment, so that no blank bottles or treatment were exposed to other iron sources.

Fresh iron oxyhydroxides were produced by spiking synthetic water samples with a 500‐mg Fe/L stock solution made with FeCl_3_·6H_2_O (97% A.C.S; Sigma‐Aldrich) to reach a nominal concentration of 1.2 mg/L. Stock solutions were prepared on the same day the experiment commenced, and the reagent was dissolved in a 0.05 M HNO_3_ solution (made with Milli‐Q water) to avoid the formation of colloidal iron before the spike was introduced to the medium. The pH of the stock solution was 1.34 ± 0.01, such that the major species present were expected to be the hexa‐aquo complex (here referred to as Fe^3+^ for simplification) and soluble iron complexes with chloride and nitrate anions (Collins et al., 2016). Commercially available iron oxyhydroxide powder (A16267, 99%+; Alfa Aesar®) was used to recreate the mineralized suspended particulate fraction in turbid waters. The iron content of this product was 65.9% w/w according to manufacturer's certificate of analysis. This powder mix was selected to allow for clear comparisons between extractions made on fresh precipitates and other iron phases with higher crystallinity typically present in iron‐rich sediments. The mineralogy of this powder was compared with that of iron‐rich sediment samples from the Hamersley Range area, in Western Australia (Supporting Information, Table S1) to verify the environmental relevance of the powder mix. These field samples were sieved in a Fritsch analysette 3 PRO shaker to recover the <63‐μm fraction, which is considered to be potentially bioavailable in sediments (ANZG, 2018), then further milled with ethanol in a Glen Creston mill to ensure a suitable crystallite size for subsequent analysis of the mineralogy by X‐ray diffraction (XRD). All samples were mounted on a front‐loaded sample holder and scans were performed in a Bruker D4 Endeavour with Co‐K*α* source and a position‐sensitive LynxEye detector, scanning 2θ from 5° to 80° with a 0.02 step‐size. Diffraction patterns were assessed with Match!® software, and mineralogy was specified by comparing to the Powder Diffraction File™ 2021 (PDF) reference database.

The formulation of the synthetic water was intended to emulate the average conditions of freshwater bodies with and without high content of suspended solids and a composition rich in mineralized iron phases naturally occurring in the sediments. The implemented formulation was a modified version of the USEPA (2002) method (Section 7), adjusted to resemble the desired water conditions for the experiment. The ionic balance calculation was performed as indicated in APHA (2017) Method 1030E. The physicochemical parameters for the formulation of the base synthetic medium were pH = 7.73 ± 0.01, hardness = 76.4 mg CaCO_3_/L, and dissolved organic carbon (DOC) = 3.85 mg/L (determined by the addition of fulvic acid). The pH was measured with a TPS WP‐80 pH‐meter and Ag/AgCl electrode calibrated with ACR pH 4 and 10 buffer solutions. To maintain stable incubation pH conditions, 3‐(*N*‐morpholino)propanesulfonic acid (MOPS; 99.5%; Sigma‐Aldrich) buffer was selected on the basis of its p*K*a value, and because it has been shown not to interact significantly with iron in solution (Hiemstra et al., 2019). The MOPS buffer was weighed and added during the formulation of the base synthetic medium to reach a concentration of 2.5 mM, but it was not included in the calculation of DOC, given the low interaction in comparison with other organic carbon sources. Hardness was calculated from calcium and magnesium determinations from inductively coupled plasma mass spectrometry (ICP‐MS; APHA 2340B) as detailed below in this section. Suwannee River fulvic acid Type III from the International Humic Substance Society was used to make a fresh 500 mg/L stock solution in Milli‐Q water from which an aliquot was added to the synthetic water formulation to reach the desired concentration. This fulvic acid type has been extensively used in iron studies, and is the type of dissolved organic matter that provides the major iron‐binding capacity in natural waters (e.g., Fujii et al., 2008). For the treatments with suspended particulate material, the nominal total suspended solids (TSS) were 80.0 mg/L, resulting in an estimated iron content of 52.7 mg/L (i.e., 65.9%). Total suspended solids were added to the synthetic water formulation by taking aliquots of a 3 g/L powder suspension in Milli‐Q water. Before each addition the suspension was vigorously mixed, and aliquots were immediately taken after refluxing the pipette tip three times to maintain an even distribution of particles in the suspension. The TSS were verified gravimetrically following APHA method 2540D from separate replicates made for this purpose (see *Results* section).

#### Experimental design

Synthetic water samples were prepared for each separate extraction experiment in the following treatment categories: blanks, single‐phase treatments, and combined‐phase treatments, each with the following composition. 1) Blank treatment: replicates with synthetic water without spiked or particulate iron. 2) Single‐phase treatments: replicates with freshly precipitated oxyhydroxides (Fe^3+^ spike to final concentration of 1.20 mg Fe/L) denominated as spiked‐only treatment, and replicates with well‐mineralized iron suspended solids in synthetic water (80 mg iron oxides/L) denominated as particulate‐only treatment. 3) Combined‐phase treatment: replicates with a mix of freshly precipitated oxyhydroxide and well‐mineralized suspended solids (1.20 mg Fe/L + 80 mg iron oxides/L).

Each treatment condition was prepared in triplicate in 250‐ml wide‐mouth, low‐density polyethylene bottles with linerless leakproof caps. To test for the effects of aging, this system of synthetic waters was replicated four times, and each of these replicate batches was incubated for 1, 3, 7, and 14 days under constant mixing on a Ratek platform mixer to keep the particulate fraction suspended. Incubation was carried out at room temperature in a temperature‐controlled laboratory (20 ± 3 °C). Each aging group comprised a batch of 12 synthetic water samples representing four possible scenarios where iron(III) is present in different forms.

To study the selectivity of different iron extraction methods, two standardized and well‐known methods implemented by commercial laboratories for the determination of trace metals as well as two cold acid extractions were applied. 1) Total recoverable metal: aliquots were analyzed following USEPA Method 200.8 (1994). 2) Filtered metals analysis: aliquots were filtered through 0.45‐μm pore size filter USEPA (1994) Method 200.8 (Section 3.3). 3) pH 2 extractable metals: water samples were acidified with HNO_3_ to pH 2 and aliquots were filtered at different time intervals for up to 16 h. 4) pH 4 extractable metals: water samples were acidified with HNO_3_ to pH 4 and aliquots were filtered at different time intervals for up to 16 h.

#### Sample processing procedure

For each of the cold‐acid extraction experiments, after each specific incubation time (i.e., aging period), a batch of 12 replicates was retrieved and the extraction assay was started. The procedure consisted of taking 10‐ml aliquots for filtered iron by filtering them through Millex‐HP polyethersulfone 0.45‐μm syringe filters. The filtrate was stored in 10‐ml polycarbonate containers and acidified to 1% v/v HNO_3_ (Sigma Suprapur). Following this, each whole replicate was processed by acidifying with 50% v/v HNO_3_ to the corresponding extraction pH (i.e., pH 2 or 4). The extraction was carried out in the same replicate bottle to avoid the loss of iron fractions adsorbed to the wall of the container. The experiment was conducted at room temperature in an air‐conditioned laboratory (20 ± 3 °C) for up to 16 h, and 10‐ml filtered aliquots were collected at sampling times 0.5, 2, 4, 8, and 16 h. The pH of the sample was monitored and replicates were thoroughly mixed before aliquots were filtered at each collection time. If a particular replicate experienced a pH drift larger than 0.03 units, dropwise corrections were made with a micropipette using 2 M HNO_3_/NaOH solutions. After 16 h of extraction time, the remaining unfiltered water sample in each replicate was mixed and transferred to a polycarbonate container for determination of total‐recoverable iron. Both pH 2 and 4 extractable and total‐recoverable samples were acidified to 1% HNO_3_. Acid blanks were collected for the purpose of controlling sample contamination and baseline correction calculations. Total‐recoverable iron digestion was performed in a Hot Block 200 (John Morris). Synthetic waters samples were digested in Environmental Express homopolymer polypropylene 50‐ml tubes with reflux caps, and final digests were diluted at the time of analysis (factor 1:10). Determinations for iron, calcium, and magnesium (for hardness calculation) were carried out in a Perkin Elmer NexION 300D ICP‐MS with a seaFAST autosampler in kinetic energy discrimination (KED) mode at the Environmental Analysis Laboratory, Southern Cross University (Lismore, Australia). For calibration, Choice Analytical certified multi‐element standards were used to develop a curve in the range 0.010–50 mg/L. The limit of detection (LOD) for iron was 0.005 mg/L.

#### Iron reactivity model

In addition to testing the ability of the extraction methods to recover the bioavailable iron fraction, it is valuable to draw possible interpretations from the results of the extraction methods with respect to physicochemical characteristics such as the initial rate of dissolution. This rate can be used as a proxy for the reactivity of iron colloids and mineralized phases. Estimations of reactivity not only allow for the statistical comparison among treatments and age groups, but also enable possible comparisons with other authors' reported values with respect to iron species. For this purpose, a model able to describe the dissolution process of iron oxyhydroxides was implemented. Christoffersen et al. (1978) described a model for which the solution composition remains constant in the following expression:

(1) $$\frac{J}{m_{0}}=k\prime {\left(\frac{m}{m_{0}}\right)}^{\gamma }$$

The model describes the mass‐normalized overall dissolution rate *J*/*m*_0_ as a function of the rate constant *k*′, and the exponential function (*m*/*m*_0_)^*γ*^ representing the proportion of remaining undissolved solid. The parameter *γ* shapes the function, modulating the mass ratio with respect to changes in the system dispersity (i.e., distribution of morphologies, active‐site density, and size distribution). The model contemplates isotropically dissolving particles which would yield values for *γ* = 1. It is expected that naturally occurring particles would not meet this condition, which led Larsen & Postma (2001) to integrate Equation 1 for *γ* ≠ 1 and formulate an expression to determine the parameters *k*′ and *γ* when the ratio *m*/*m*_0_ is plotted as a function of time:

(2) $$\frac{m}{m_{0}}={\left[-k\prime \left(1-\gamma \right)t+1\right]}^{\frac{1}{1-\gamma }}$$

By fitting Equation 2, it is possible to calculate the parameter values necessary to determine the overall rate constant as a function of the calculated proportion of undissolved solid. Furthermore, by plotting −log(*J*/*m*_0_) versus −log(*m*/*m*_0_) we obtain a straight line with slope = *γ*. This allows for the graphical comparison of the initial rate constant *k*′ when *m*/*m*_0_ = 1 (i.e., initial conditions) of different iron phases, making the comparison of different reactivities an intuitive exercise. The fraction *m*/*m*_0_ was defined as the final acid‐recoverable determination so that the calculated reactivity values only represent the reactive iron pool (Hyacinthe et al., 2006).

### Statistical analysis

Statistical analysis was performed using R software (R Core Team, 2022). Nonlinear least‐squares (NLS) fitting was coded with *nls* function and starter values were selected based on the literature and by visual graphic approximation. Nonlinear model parameters were extracted from the model matrix and their corresponding standard errors and 95% confidence intervals were calculated by applying the *sandwich* package to control for autocorrelated and heteroscedastic errors. because *R* ^2^ values are unreliable goodness‐of‐fit indicators for nonlinear models (Kvålseth, 1985), model fitness was assessed via Neill's test (Neill, 1988). Model coefficients were contrasted by *t*‐test or by a nested model approach using an *F*‐test (Ritz & Streibig, 2008). Cross‐sectional assessment of the time series was performed with weighted linear regression and one‐way analysis of variance (ANOVA) using the packages Metafor and Car. In all cases, normality assumptions were evaluated with a Shapiro test for the model residuals and via visual inspection in *qq*‐plots. Data log‐transformation was applied for cross‐sectional analysis when needed, in which case only relative change was reported. All data‐related figures were produced with the ggplot2 and cowplot packages.

## RESULTS

The XRD analysis indicated the presence of iron minerals, including hematite, magnetite, and goethite, in all samples. Spectra from natural sediments also showed marked peaks for quartz, but mostly resembled the diffraction pattern of the synthetic powder mix (Supporting Information, Figure S1). Gravimetric determination of suspended solids yielded a mean concentration of 76.0 mg/L (SD 2.87).

All blanks processed for filtered iron had concentrations close to (range 0.005–0.007 mg/L) or below the LOD (<0.005 mg/L). The pH 2 extractable iron fraction in blanks was below the LOD (<0.005 mg/L) after 16 h, and the total‐recoverable fraction of blanks in the pH 2 experiment yielded values one order of magnitude larger (*X̄* 0.05 mg/L, SD 0.03; more details can be found in Supporting Information, Table S2). The pH was maintained at 2 ± 0.02 units throughout the pH 2 experiment.

The pH 4 extractable iron concentrations in blanks ranged from <0.005 to 0.006 mg/L, and the concentrations of total‐recoverable iron in blanks of the pH 4 experiment averaged 0.03 mg/L (SD 0.01) after 16 h (Supporting Information, Table S4). The pH was maintained at 4 ± 0.02 units throughout the experiment.

The total‐recoverable fraction of the spiked‐only treatment in the pH 2 experiment was 1.20 mg Fe/L and for the pH 4 experiment it was 1.17 mg Fe/L. For the particulate‐only and combined treatments, the total‐recoverable iron determinations for the pH 2 experiment were 28.2 and 32.0 mg/L, respectively (Supporting Information, Table S3). On the other hand, in the pH 4 experiment, the total‐recoverable iron fraction in the particulate treatment was 31.9 mg/L and for the combined treatment was 36.3 mg/L (Supporting Information, Table S5). The average recoveries considering both experiments were 56.3% and 63.7% with respect to the iron content in the added suspended powder mix (nominal 50.1 mg Fe/L) for the single treatments. This low recovery may reflect the method's performance limitations toward high content of mineralized phases. A further digestion in HNO_3_ showed the mean iron concentration was 44.1 mg/L (SD 2.02); a mean recovery of 88.1% with respect to the nominal concentration. Another contributing factor may have been the loss of some mass due to adsorption of fine particulates to the incubation bottles, which was experimentally and partially minimized by working with large volumes (i.e., 1 L in 250‐ml replicates) and pre‐wetting the iron powder by making a suspended stock. Lastly, the variation in total‐recoverable iron in treatments with added TSS could also be due to the difficulties in maintaining a perfectly homogenized sample at the time of taking an aliquot for the digestion in the hot‐block step.

In terms of recovery, the spiked‐only replicates showed that average filtered‐iron values from both experiments were <0.50 mg/L, only representing up to 40.5% of the spiked iron (1.20 mg/L) depending on the aging group (Table 2). These samples also showed high variability within and among the different aging groups, with no clear trend or significant effect due to aging detected by ANOVA analysis (*F* = 0.38, *p* = 0.77). Filtered‐iron values in the particulate‐only treatments were ≤0.01 mg/L and neither reflected the effects of aging (*F* = 0.84, *p* = 0.49). As for the combined treatments, values for filtered iron were similar to those in the spiked‐only treatment and did not show differences with respect to incubation time (*F* = 0.36, *p* = 0.76). Given that the filtered iron recovery of the particulate‐only treatments was very low, the recovery observed in the combined treatment would be below 40% of the spiked iron (Table 2).

**Table 2:** Mean iron concentration, standard deviation (SD), and recovery (%) for samples processed by filtration only (0.45 µm) of all aging groups in both experiments

Fraction	Treatment	Age (days)	*X̄* (mg/L)	SD (mg/L)	Recovery (%)
Filtered	Single spiked	1	0.49	0.20	40.5
		3	0.38	0.20	31.5
		7	0.38	0.27	31.5
		14	0.38	0.16	31.9
	Single particulate	1	0.01	0.02	1.15
		3	0.01	0.004	0.38
		7	0.01	0.01	0.63
		14	0.01	0.01	0.77
	Combined	1	0.43	0.19	35.9
		3	0.36	0.31	29.8
		7	0.37	0.26	31.0
		14	0.29	0.19	23.8

Recovery was calculated with respect to the total recoverable fraction.

**Figure 1:**
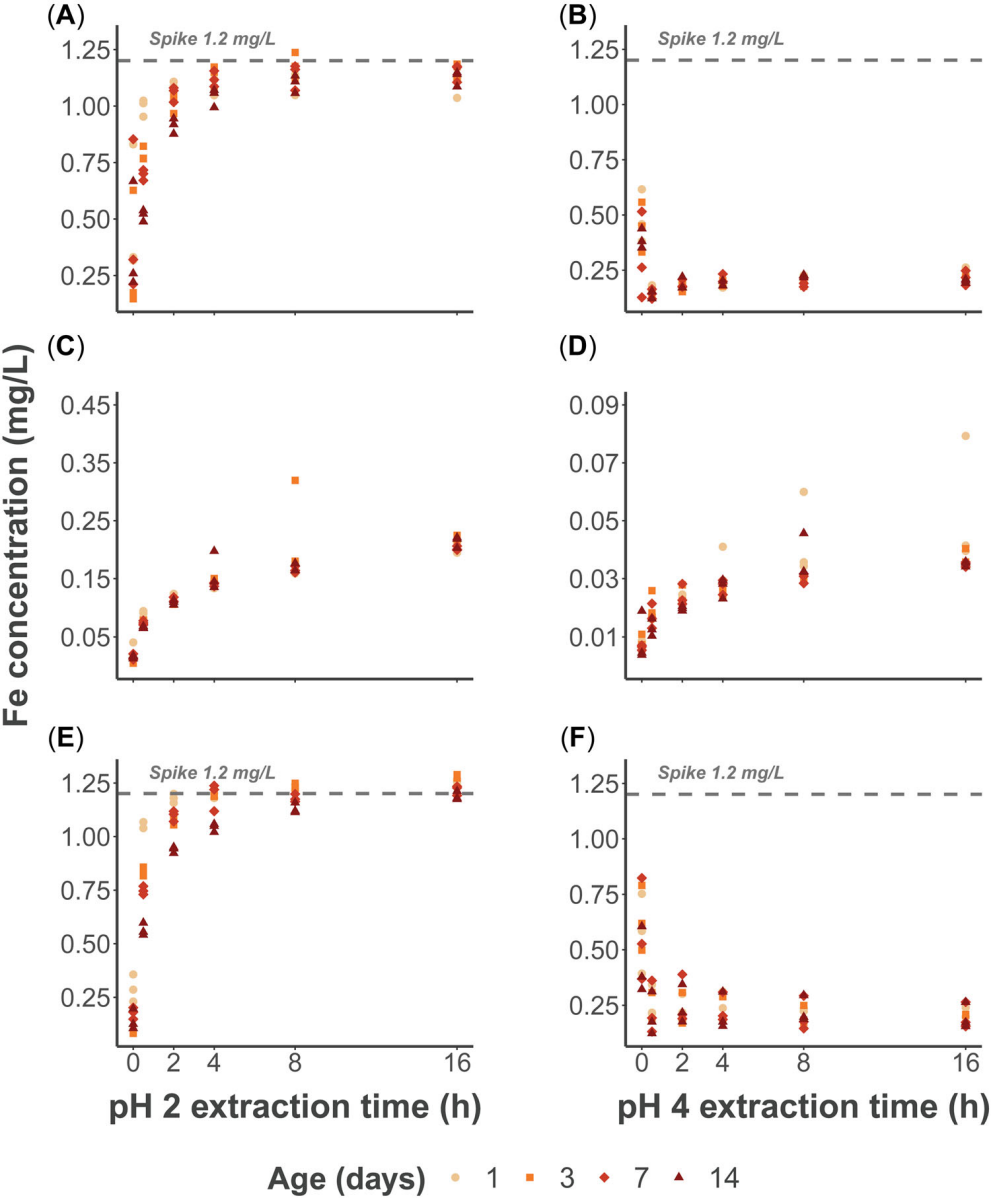
Evolution of the iron dissolution for each aging group as a function of time (h) during the pH 2 and pH 4 extractions in the spiked‐only (**A** and **B**), particulate‐only (**C** and **D**), and combined (**E** and **F**) treatments.

Overall, the pH 2 extractions showed similar dissolution behavior in the spiked‐only and combined treatments for each aging group, whereas the particulate‐only data followed a slower and steadier trend (Figure 1; Supporting Information, Table S3). Samples in the pH 4 experiment showed very low recoveries in the two treatments with added iron spike. The particulate‐only results followed a similar trend to that of the pH 2 extraction, but displayed greater variability (Figure 1 and Supporting Information, Table S5).

**Figure 2:**
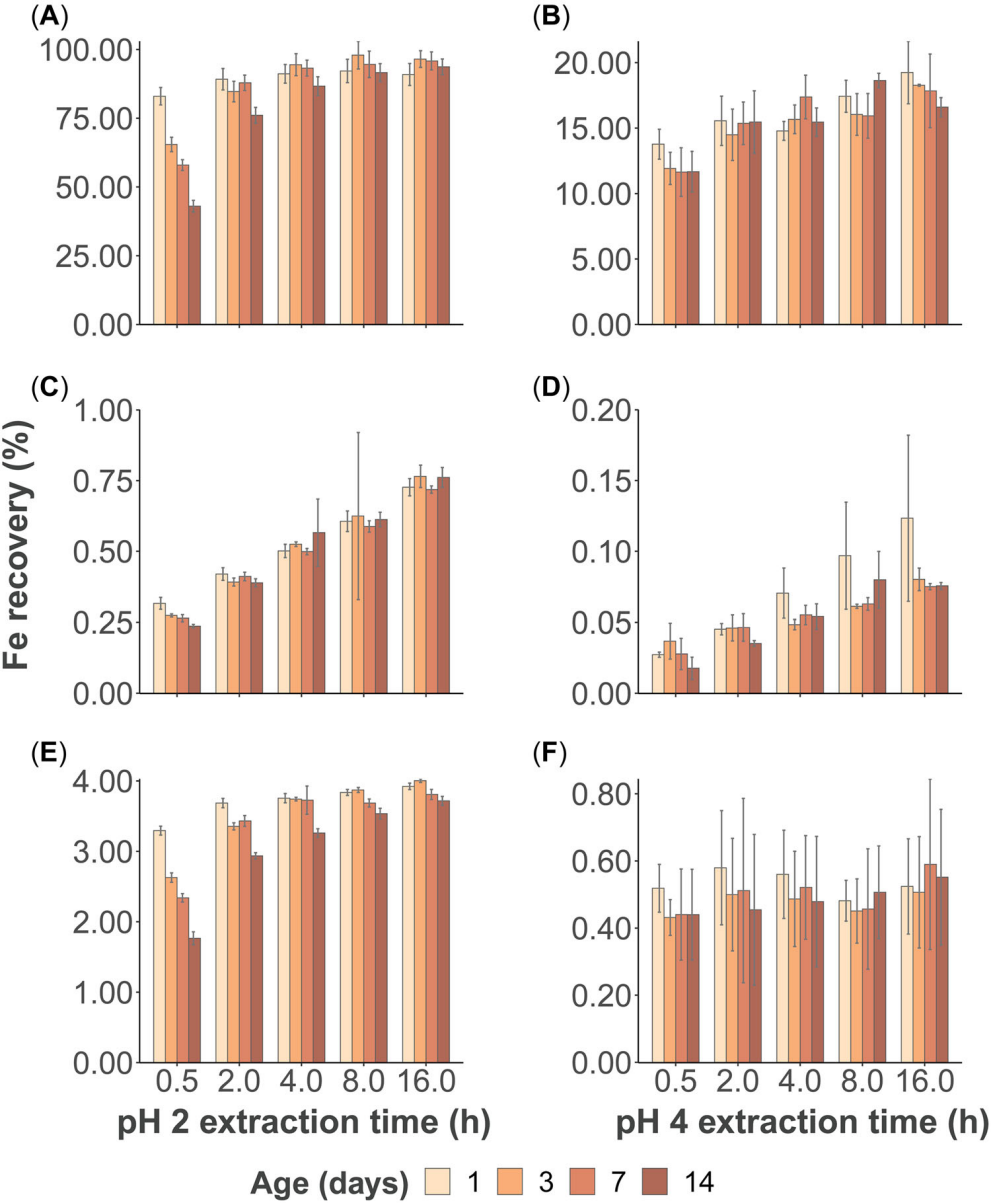
Iron recovery (%) for each aging group and extraction time (h) in the spiked‐only (**A** and **B**), particulate‐only (**C** and **D**), and combined (**E** and **F**) treatments during the pH 2 and 4 experiments. Exact values are available in the Supporting Information.

The pH 2 extraction in the spiked‐only treatment showed that recoveries for all samples increased toward a maximum value with time, approaching a maximum value after a sampling time of 4 h. Recoveries of all samples after the standard 16 h were 91%, 97%, 96%, and 94% of the total‐recoverable fraction (1.20 mg/L) for 1, 3, 7, and 14 days of aging, respectively (Figure 2A). Furthermore, pH 2 extractable iron was recovered above 90% for most aging periods at extraction times higher than 4 h, with the exception of the 14‐day incubation replicates (87%; Supporting Information, Table S6). The sampling times which showed the best discriminating resolution among samples of different ages were 0.5 h (*F* = 131.88 *p* < 0.001) and 2 h (*F* = 9.25 *p* < 0.05), but more conspicuously for the former sampling time. No discriminating resolution among aged samples was detected for a sampling time of 16 h (*F* = 1.74, *p* = 0.24; Supporting Information, Table S7).

For the particulate‐only treatment, the pH 2 extractable fraction represented <1% of the total recoverable fraction (28.2 mg Fe/L; Figure 2C and Supporting Information, Table S8). At sampling time 0.5 h, the pH 2 extraction showed significant differences among the aged samples (*F* = 20.0, *p* < 0.001; Supporting Information, Table S7). In the case of the combined treatment, the pH 2 extractable fraction responded in a similar fashion to the spiked‐only treatment (Figure 2E and Supporting Information, Table S9). Significant differences were found at all extraction times. However, the 0.5‐h sampling time showed the largest differences in mean recovered iron (*F* = 187, *p* < 0.001) among age groups, implying a better discriminating power (Supporting Information, Table S7).

The pH 4 extraction did not show an adequate performance in recovering the added iron spike. In the spiked‐only treatment, recoveries were below 20% after 16 h (Figure 2B and Supporting Information, Table S10). Moreover, despite the similar dissolution trend observed in the particulate‐only treatment in comparison with the pH 2 replicates, iron concentrations for a sampling time of 16 h were an order of magnitude lower and comparable to the range yielded for 0.5 h of the pH 2 extraction of the particulate‐only treatment (Figure 2D and Supporting Information, Table S11). In the same fashion, the combined treatment resulted in low recoveries (<1%) after 16 h of the pH extraction. Overall, the pH 4 extraction in the combined treatment yielded iron values similar to those achieved after 16 h of pH 2 extraction in the particulate‐only treatment (Figure 2F and Supporting Information, Table S12). No significant differences were detected for the effects of incubation time (*p* > 0.05) for all sampling times and treatments during the pH 4 extraction (Supporting Information, Table S13).

Due to the low recovery and the lack of discrimination of different aged samples observed in the pH 4 extraction, the Larsen & Postma (2001) model was only fitted to the pH 2 extraction data. Nonlinear model fits from the spiked‐only dissolution data complied with the normality assumptions for the residuals and were all satisfactory, with no rejection of the Neill's test null hypothesis (*p* > 0.05; Table 3 and Supporting Information, Figure S2A).

**Table 3:** Specifications of the nonlinear regression model fit for each aging group in the spiked‐only treatment

Treatment	Aging time (days)	Shapiro test (*p* value)	Neill's test (*F*)	Lack of fit (*p* value)	*k*′ (s^−1^)	*k*′ (SE)	*γ*	*γ* (SE)
Single spiked	1	0.61	1.09	0.40	5.41 × 10^**−**3^	1.21 × 10^**−**3^	2.05	1.2 × 10^**−**1^
	3	0.98	0.60	0.67	1.04 × 10^**−**3^	1.85 × 10^**−**4^	1.94	1.9 × 10^**−**1^
	7	0.10	0.17	0.95	6.47 × 10^**−**4^	1.16 × 10^**−**5^	1.47	4.28 × 10^**−**2^
	14	0.36	1.19	0.36	4.02 × 10^**−**4^	1.05 × 10^**−**5^	1.57	3.84 × 10^**−**2^

Estimated parameters *k* and *γ* are accompanied by their respective standard errors (SE).

**Figure 3:**
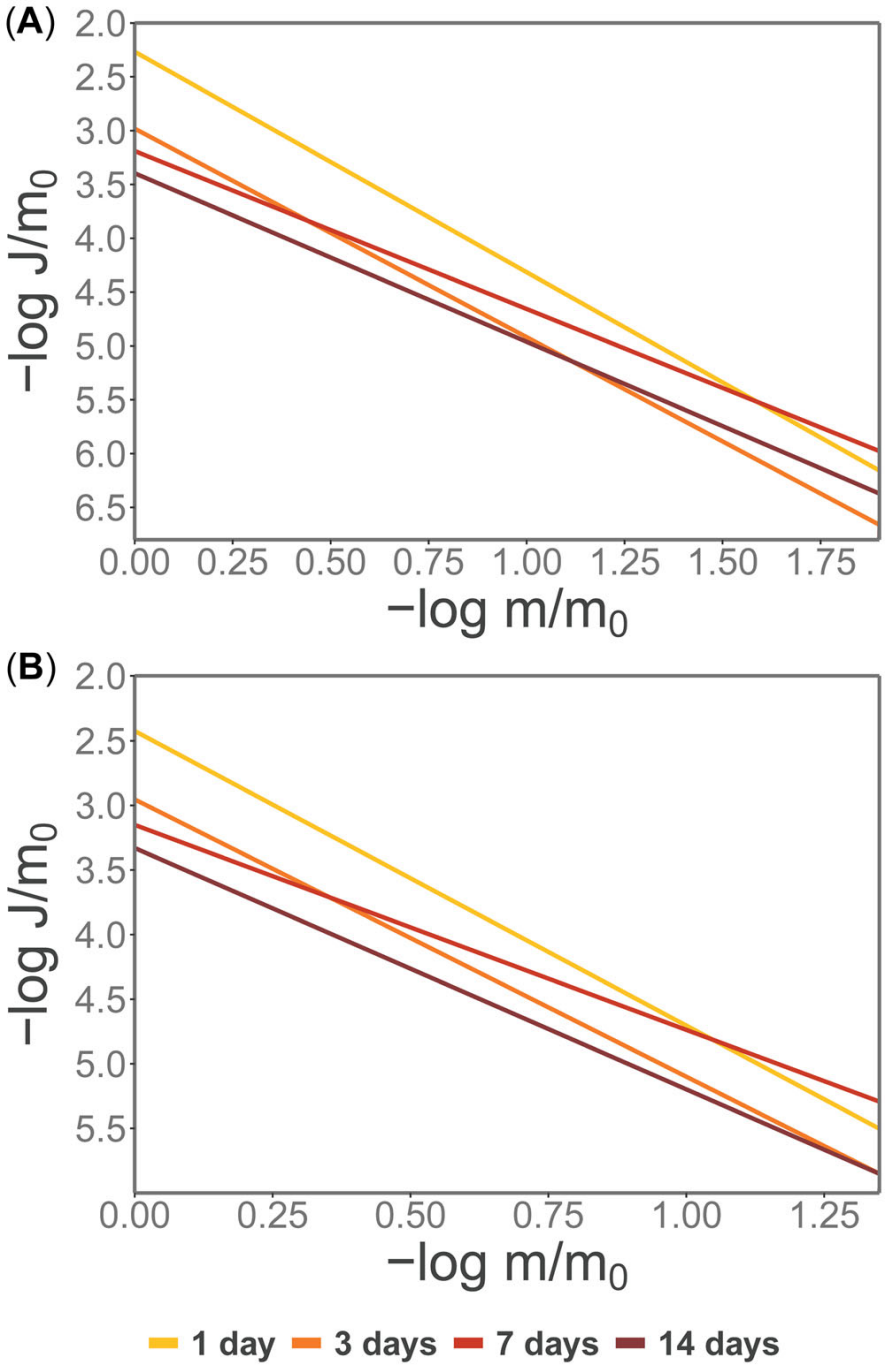
Linearized representation of the change of overall dissolution rate in each aging group as a function of the remaining solid phase in the spiked‐only (**A**) and combined (**B**) treatments during the pH 2 extraction experiment.

A −log(*J*/*m*_0_) versus −log(*m*/*m*_0_) plot showed the normalized overall dissolution rate against the proportion of undissolved iron as a straight line with slope corresponding to parameter *γ*, which at initial conditions −log(*m*/*m*_0_) = 0, the intercept equals the initial rate constant *k*' (Figure 3). The assumption for the initial condition was derived from the fact that under the incubation conditions, and due to the observed effect of aging on the recovery rates, the majority of the spike was likely present as polymeric species and iron oxyhydroxides (see *Discussion* section). This graphical representation of the initial reactivity of the different aging groups in the spiked‐only iron showed two reactivity trend groups: one with a more rapid decrease in dissolution rate with extraction time for sample ages 1 and 3 days, and a second group with a slower decrease of the dissolution rate for samples aged for 7 and 14 days (Figure 3A). Furthermore, initial dissolution rates for the 1‐ and 3‐day‐old samples were an order of magnitude higher than those for samples aged for 7 and 14 days (Figure 3A). This grouping was statistically supported, with no significant differences (*p* > 0.05) detected between ages 1 and 3 days, nor between ages 7 and 14 days. Although the dissolution rate for the precipitates aged for 3 days followed the same trend as 1‐day‐old samples, the rates became similar to those of the 7‐ and 14‐day‐old samples at early and later stages of the dissolution process, respectively. A similar trend was observed for 1‐day‐old samples, which exhibited equivalent dissolution rates similar to those of the samples aged for 7 days (Figure 3A). The cross‐sectional log‐transformed weighted linear regression for the initial rate constants showed significant effects exerted by the aging process, with a relative decrease in rate constant value of 15.1% for the back‐transformed values (Supporting Information, Table S14).

Data from the combined‐treatment samples were fitted to the model and parameters were successfully estimated (Supporting Information, Figure S2B). The results showed that values for *k*' and *γ* were within the same range as those for the spiked‐only treatment (Table 4). The linearized model showed similar trends to those observed in the spiked‐only treatment, with samples aged for 1 and 3 days having similar slopes (*p* > 0.05) and following a distinctive common trend to that of samples aged for 7 and 14 days (Figure 3B). Intersection of the linearized model for different sample ages occurred, holding the same pattern as for the spiked‐only treatment. However, this time the intersection points took place at stages where dissolution had not consumed as much precipitate as in the spiked‐only treatment. This not only suggests an increasingly stable nature of the remaining undissolved phase (potentially due to interactions with the particulate fraction), but also it is in line with the hypothesis that iron was present as precipitates subjected to aging processes. Notwithstanding this, it is worth noting that other processes such as the coprecipitation of other cations or the stabilizing nature of organic matter could be contributing to the increasing stability of the precipitates. These, however, would have manifested in the spiked‐only treatment as well. A comparison by treatment of the nonlinearized models can be observed in Supporting Information, Figure S3.

**Table 4:** Specifications of the nonlinear regression model fit for each aging group in the combined treatment

Treatment	Aging time (days)	Shapiro test (*p* value)	Neill's test (*F*)	Lack of fit (*p* value)	*k*′ (s^−1^)	*k*′ (SE)	*γ*	*γ* (SE)
Combined	1	0.20	0.73	0.59	3.77 × 10^**−**3^	5.47 × 10^**−**4^	2.28	0.10
	3	0.03	3.14	0.06	1.11 × 10^**−**3^	1.03 × 10^**−**4^	2.15	0.09
	7	0.10	0.69	0.61	7.07 × 10^**−**4^	4.58 × 10^**−**5^	1.59	0.08
	14	0.18	1.47	0.27	4.68 × 10^**−**4^	2.18 × 10^**−**5^	1.87	0.06

Estimated parameters *k* and *γ* are accompanied by their respective standard errors (SE).

Although the initial rate constant for the spiked‐only treatment seemed to be higher than for the combined treatment, the contrast of coefficients was not significant (*p* > 0.05). Although this apparent difference might be possible within the intrinsic variability associated with the experimental errors, in a further statistical assessment an *F*‐test for the 1‐day age fit of the pooled data (spiked + combined treatments) with shared parameters versus separate parameters showed significant differences (*F* = 54.464, *p* < 0.001), suggesting the overall data behave differently enough to require two different models to describe them (Supporting Information, Table S15). These differences were also detected among the 14‐day data, whereas comparisons of dissolution of the 3‐ and 7‐day‐old samples were not significant (Supporting Information, Table S15). It is possible that these differences reflect the underlying kinetics of dissolution of the well‐mineralized particulate phase in the combined treatment data. Unfortunately, the experimental design did not accommodate delving into the exact discrimination of the provenance of the extracted iron comprising the recovered fraction. This apparent contribution from the coexisting well‐mineralized particles seems, from a methodological perspective, to be negligible (i.e., no interference with the recovery of the spike).

The weighted one‐way ANOVA of the log‐transformed data showed significant effects of the aging process on the initial rate constant, with a relative decrease in the rate constant value of 12.6% for each day of aging, a smaller effect with respect to the spiked‐only treatment but nonetheless statistically significant for the contrast of coefficients (*t* = 9.36, *p* < 0.001; Supporting Information, Table S16).

Model fits of the data from the treatment with the particulate fraction were relatively poor, with a persistent overprediction of the extracted fraction in the early stages of the dissolution and consistent underprediction for the final stage of the dissolution process for all sample ages (Supporting Information, Figure S4). On further exploration, it was possible to obtain better fits for the first 4 h of the dissolution curve (Table 5 and Supporting Information, Figure S5). To ensure comparability, trimmed data from the spiked‐only and the combined treatments were fitted and contrasted (via *F*‐test) to their respective full‐data models fits. For all sample ages from the spiked‐only and combined treatments, there were no significant differences (*p* > 0.05) in the estimated parameters, indicating that the full model could be used instead of the trimmed data fits. This was not found for the particulate‐only treatment model fits, which exhibited significant differences between parameters of trimmed‐data fits and full‐data fits (*p* < 0.001). Details for the different model contrasts can be found in Supporting Information, Table S17. Comparisons of the initial dissolution rates between the particulate‐only and the spiked‐only and combined treatments show that, for the well‐mineralized fraction, reactivities are within the same order of magnitude observed in samples with added Fe‐spike which had been incubated for at least 7 days. Values for *γ* were higher than those for the combined and spiked‐only treatments, suggesting a greater variation in the dispersity of the powder mix. This agrees with the fact that the powder mix was a combination of hematite, magnetite, and goethite particles (Supporting Information, Figure S1).

**Table 5:** Specifications of the nonlinear regression model fit for each aging group in the particulate‐only treatment

Treatment	Aging time (days)	Shapiro test (*p* value)	Neill's test *F*	Lack of fit (*p* value)	*k*′ (s^−1^)	*k*′ (SE)	*γ*	*γ* (SE)
Single particulate	1	0.87	1.71	0.24	9.73 × 10^**−**4^	2.39 × 10^**−**4^	4.81	0.47
	3	0.35	2.39	0.15	4.25 × 10^**−**4^	6.14 × 10^**−**5^	4.04	0.37
	7	0.12	0.88	0.45	4.58 × 10^**−**4^	4.51 × 10^**−**5^	3.64	0.20
	14	0.59	1.83	0.22	3.11 × 10^**−**4^	3.19 × 10^**−**5^	3.38	0.22

Estimated parameters *k* and *γ* are accompanied by their respective standard errors (SE).

## DISCUSSION

Assessing iron bioavailability requires a good understanding of its speciation. A wide range of techniques has been developed to sample and preserve chemical species for laboratory analysis (Batley, 1989; Hopwood et al., 2014). From a research perspective, introducing bioavailability in the risk assessment process involves the interpretation of numerous variables and focused approaches, which demands time and resources to develop predictive models. These models, although sophisticated and effective, may be difficult to implement in environmental management schemes due to cost and logistical constraints. It is therefore desirable to develop tools which introduce an adequate degree of chemical specificity yet are simple enough to be incorporated into ongoing and already established regulatory schemes (Merrington et al., 2016). In this investigation, a suite of well‐established and standardized methods was tested to assess their suitability to operationally define iron fractions of higher bioavailability in freshwater systems with a high content of suspended particulate material.

The standard method for determination of dissolved iron involves the filtration of water samples through a 0.45‐µm pore size filter and the subsequent acidification of the sample for preservation until chemical analysis takes place. However, soluble iron is quite scarce and due to its natural tendency to hydrolyze it is mostly present in precipitated form in circumneutral oxygenated waters. In the different experiments presented in our study, filtered iron fractions showed low recoveries with respect to the added iron spike. In the case of the particulate‐only treatment, recoveries were below 0.1% of the total recoverable iron. This result is not surprising due to the low solubility of the well‐mineralized iron oxides forming the suspended particulate fraction. However, filtered iron was higher than in the blank treatment, showing that despite being trapped by the filter membrane, certain particles made it through the filter. This is supported by the well‐known issues of nanoparticulate iron passing through membranes and inflating solubility limit calculations (Fox, 1988). On evaluating the performance of the filtered fraction to discriminate the effects of aging on the different treatments, it was evident that no specific trend occurred in any of the treatment groups (Table 2). One of the main drawbacks of operationally defined fractions based on filtration techniques is the insensitivity with respect to adsorption processes. If a target chemical species is adsorbed to the particulate fraction, and the particles are large enough to be removed by a filter membrane, then the adsorbed species will be under‐represented (Davison & De Vitre, 1992; Lead et al., 1997). This was observed for the pH 2 extractable fraction in the combined treatment, which in comparison with the filtered recovery accounted for more iron than that expected for the particulate fraction only, hence confirming the spike presence in the system. The Appendix C of the draft Australian default iron(III) guideline value document (temporarily released for public consultation) specifically addresses this as a concern. These results support the understanding that potentially bioavailable colloidal iron may be removed from samples by filtration devices and lead to false negative results with respect to the guideline value (ANZG, 2020).

Lastly, operationally defined dissolved iron has been found to correlate strongly with DOC levels in natural waters (Allard et al., 2011; Fujii et al., 2008), but the complexation of iron by DOC is associated with lower bioavailability (Arbildua et al., 2017). Complexed soluble iron can be recovered by other methods such as the acid‐soluble method applied in these experiments. Fujii et al. (2008) showed that a short (1‐min) acidification step to pH 2 was enough to recover the organically bound soluble iron fraction from coastal waters. This could be a potential added step to account for organically complexed iron as part of a sampling scheme where the truly soluble fraction is subtracted from the >4‐h pH 2 extraction to operationally define a bioavailable iron fraction in freshwaters. Other rapid methods involving ion exchange resins have been tested and shown to allow for separation of the soluble fraction without interference of colloidal iron (Bowles et al., 2006). Alternatively, the method could be implemented with a conservative approach by including the soluble Fe–NOM fraction because, in geochemical terms, that fraction is one of the many responsible for maintaining a steady‐state source of iron for the cycling of freshly formed colloidal precipitates in the water column. In our experiments, recoveries for filtered iron may have included fractions of soluble Fe–NOM complexes. These are well known to stabilize iron against hydrolysis (Karlsson & Persson, 2012), but extended X‐ray absorption fine structure analyses have also shown that both monomeric and polymeric species as well as ferrihydrite are present in association with NOM (Sundman et al., 2013). Moreover, Fujii et al. (2008) showed that although soluble organically‐bound iron is rapidly released after pH 2 acidification, NOM–Fe_*n*_(OH)_*m*_ complexes are only released slowly due to the changes induced by aging mechanisms. However, the clear decrease in the recovery of iron throughout the extraction period with different incubation times can be primarily attributed to the effects of aging on the reactivity of iron colloids. Furthermore, the average half‐life for Fe–NOM complexes was calculated to be 14 days (Fujii et al., 2008). If Fe^3+^ had been present, this would have manifested in the later incubation days as calculated rate constants in the same or higher range than the younger precipitates, but this was not observed. Finally, the dissolution model showed dissolution rate trends with a clear chronological pattern for incubation times, rendering older samples less reactive than the younger ones, therefore values of filtered iron likely originated from the presence of smaller particles of iron hydroxides as opposed to truly soluble iron.

The USEPA standardized method for acid‐soluble metals uses a 16‐h total extraction time at pH 1.75, after which 0.45‐μm filtration is applied and samples are acidified and analyzed (USEPA, 1991). The pH 2 and pH 4 extractions involved following the dissolution process at five different times. After 16 h, the pH 2 extraction showed contrasting results between treatments with different iron sources. Less than 1% iron recovery of the well‐mineralized powder mix is consistent with the increased thermodynamic stability of the iron oxyhydroxide crystal structure (Cornell & Schwertmann, 2003). Cold‐acid digestion is only able to detach surface iron atoms at a very slow rate (Sulzberger et al., 1989). Surprisingly, the pH 2 extraction with 0.5 h sampling time still showed significant differences among the different incubation times in the well‐mineralized fraction, which showed low variation despite the low yields involved. This unexpected data pattern might be reflecting the effects of hydration on the surface of iron oxyhydroxides. Although iron oxides present particularly low solubility constants, their surface chemistry is quite active, and hydration of the outer layers of the minerals has been observed to confer higher solubilities comparable to those of goethite or ferrihydrite (Jang et al., 2007). Our results agreed with this analysis, showing initial rate constants for this labile surface species in the same order of magnitude as for precipitates aged for 7 and 14 days. This phenomenon would also be in line with reported rapid release of iron at the very beginning of the dissolution process and the subsequent deceleration with very low yields (Cornell & Schwertmann, 2003). Furthermore, on a longer environmental time scale, this agrees with the reported changes from hematite to goethite (and vice versa) in aquatic environments, particularly when dry–wet cycles take place (Guo & Barnard, 2013).

It is important to point out the observable differences in the dissolution curves of the spiked treatments and the mineralized particulates. The trend for the former turns asymptotical when it reaches values close to the added spike, whereas for the latter it shows an initial steeper trend in the first 30 min of the extraction but eventually shifts to a slower almost linear trend with no signs of plateauing (Figure 1). This kinetic behavior could be conceptualized as the combination of two different processes with different rate constants, one for the hydrated surface species and another for the denuded‐bulk hematite particle. Indeed, sorption–desorption assays seem to confirm this behavior, which has been fitted using kinetic models with more than one rate constant (Samson & Eggleston, 1998). If these observations are correct, then the cold‐acid extraction would be recovering the more reactive surface component of the iron oxides and, depending on the timing of the extraction process, a minor fraction of the mineralized bulk phase. In either case, the well‐mineralized fraction would only be extracted at lower levels with respect to freshly precipitated iron hydroxides. Notwithstanding the very low proportion of reactive iron recovered from the well‐mineralized fraction, it will be important to test this extraction in the presence of fresh field samples, which could contain iron oxides and oxyhydroxides with a greater degree of hydration and therefore a higher content of a surface reactive fraction. A pH 2 extraction of >4 h might recover this surface fraction and potentially inflate determinations of the bioavailable fraction.

The pH 2 extraction recovered most of the spike after 4 h of extraction regardless of the aging time. Aging‐sensitive properties were observed for extraction times below 4 h. The most discriminating effect was observed for the 0.5‐h treatment, with recoveries >80% of the 1‐day aged precipitates. Comparatively, the difference in recovery between the younger and older samples was larger for *t* = 0.5 h than for *t* = 4 h (spiked‐only: 40% vs. 13%, and combined: 51% vs. 10%, respectively). The observed decrease in recovery with increasing incubation times agrees with the expected effects of the aging process on the thermodynamic stability of iron phases (Cornell & Schwertmann, 2003). This decreasing trend was statistically significant, showing decreases of 15% and 12% of reactivity for every day of aging for the spiked‐only and combined treatments, respectively. The dissolution model showed two groups of reactivities, with a more labile fraction for 1‐ and 3‐day aged samples in comparison with those incubated for 7 and 14 days.

The pH 4 extraction did not show an adequate discriminating resolution with respect to aging groups and only recovered less than 25% of the added spike in both combined and spiked‐only treatments. The particulate‐only treatment showed recoveries below 0.20% of the total‐recoverable fraction. This low performance is likely related to the effects of chemi‐ and physi‐sorption observed for Fe^3+^ onto hematite (Ambe & Ambe, 1990), and the marked consequences of the onset of hydrolysis above pH 3 for iron(III) (Samson & Eggleston, 2002; Samson et al., 2000). Furthermore, the adsorption edge of Fe^3+^ on silica and aluminum oxide falls within the pH interval 2–4 (Parks, 1990). Thus, the pH 2 extraction performs better because it operates at the lower bound of the Fe^3+^ adsorption edge and in a pH range in which dissolution outcompetes precipitation.

The rapid dissolution of iron from the added spike at the beginning of the assay agrees with other published data by Bligh & Waite (2011), Jones et al. (2009), and Larsen & Postma (2001). Their *k*' values were in the range estimated by our study (10^−3^–10^‐4^ s^−1^), whereas our values for *γ* were more in line with those reported by Larsen & Postma (2001), both for dissolution of two‐line ferrihydrite. This further supports the assumption that most iron in samples was present as precipitates such as ferrihydrite, and that values of filtered iron were likely the result of nanoparticles passing through the filter membrane.

The results from the total‐recoverable determinations showed that well‐mineralized suspended particulate materials will be extracted despite their high crystallinity and lower reactivity. Furthermore, by implementing the method in a combined fraction treatment, it has been demonstrated that the total‐recoverable fraction will overestimate the amount of reactive iron in the water samples.

In defining a bioavailable fraction, it is necessary not only to establish the operational conditions to allow for the desired determinations, but also the proposed method must be contrasted against a biological response. The experiments in the present study have provided evidence of how the effects of aging impact on the reactivity of colloidal iron and have demonstrated how incubation times beyond 7 days significantly reduce the reactivity of colloidal iron to levels comparable to the minor fraction extracted from well‐mineralized iron oxides and oxyhydroxides. Understanding how these changes in reactivity correlate with the toxic response of freshwater organisms is where our efforts are currently focused. However, it is interesting to understand the potential ramifications of the findings of the present study given that toxicity data from the literature comes from a wide range of colloidal iron ages (in many cases not specified). For example, Biesinger & Christensen (1972) derived chronic 16% effect concentration values for *Daphnia magna* after exposure to 4‐day‐old iron suspensions, whereas Arbildua et al. (2017) and Sykora et al. (1972) used 3‐ and ~2‐h‐old suspensions in their experiments, respectively. Yoshida et al. (2006) demonstrated how iron(III) uptake in diatoms substantially decreased when comparing treatments with direct addition of Fe^3+^ versus iron colloids aged for 1 and 3 days, and up to 3 weeks. The 3‐week‐old treatment resulted in low uptakes close to the negative control level. Thus, an operationally defined bioavailable fraction should preferably target a certain range of reactivities and this should account for them retrospectively because sample processing times may not always be as prompt as necessary (e.g., field samples from remote areas would take a considerable amount of time until the sample is extracted in practice). Under this last scenario, one could imagine a ≥4‐h pH 2 extraction method as a good approach to recover a bioavailable fraction of iron within a 14‐day window of time. This would give commercial laboratories a reasonable amount of time to process samples. However, in a laboratory‐controlled environment, such as during the implementation of bioassays, a more instantaneous method such as the 0.5‐h pH 2 extraction method could prove to be a practical alternative, especially if toxicity is observed only in colloidal iron(III) <1 day of age. Such a rapid method could potentially be implemented in situ during field monitoring routines.

## CONCLUSION

Overall, determinations of fractions such as “dissolved Fe” (i.e., filtered), “total‐recoverable Fe,” and “pH 4 extractable Fe” were shown to be unsuitable to operationally define iron fractions of higher bioavailability in waters with mineralized suspended particulate material. Conversely, the pH 2 extractable iron fraction has been shown to not only include the more reactive fractions of iron, but also it has been demonstrated that the method can be adapted to “fine‐tune” the resolution toward specific reactivities associated to the aging process of iron precipitates. Most importantly, our experiments have shown that for unfiltered samples, pH 2 extraction times of ≥4 h recover labile particulate iron forms resulting from a 1.2‐mg/L Fe^3+^ spike addition with a negligible contribution from well‐mineralized iron oxides and oxyhydroxides present at concentrations up to 76 mg/L. It is also important to note that the question regarding whether the ≥4‐h fraction overestimates the potentially bioavailable fraction compared with what may be extracted in a shorter period (e.g., 0.5 h) needs further study, including comparison with ecotoxicological response data in synthetic and natural waters.

## Supporting Information

The Supporting Information is available on the Wiley Online Library at https://10.1002/etc.5530.

## Conflict of Interest

The authors declare no conflict of interest.

## Disclaimer

This manuscript has not been published elsewhere nor is it under consideration by another journal. All authors have approved the manuscript.

## Author Contributions Statement

**Emiliano Balsamo Crespo**: Conceptualization; Data curation; Investigation; Formal analysis; Methodology; Visualization; Writing—original draft; Writing—review & editing. **Amanda Reichelt‐Brushett**: Supervision; Resources; Conceptualization; Methodology; Validation; Writing—review & editing. **Ross E. W. Smith**: Supervision; Resources; Conceptualization; Methodology; Validation; Writing—review & editing. **Andrew L. Rose**: Supervision; Resources; Conceptualization; Methodology; Formal analysis; Validation; Writing—review & editing. **Graeme E. Batley**: Conceptualization; Methodology; Validation; Writing—review & editing.

## Supporting information

This article includes online‐only Supporting Information.

Supplementary information.

## Data Availability

The data are a part of an ongoing PhD thesis and are mostly available in the Supporting Information. The data, associated metadata, and calculation tools are also available from the corresponding author (e.balsamo.crespo.10@student.scu.edu.au).
