# Supplementary material for: Improving the Measurement of Iron(III) Bioavailability in Freshwater Samples: Methods and Performance
Source: Environ Toxicol Chem. 2022 Dec 20;42(2):303–16. doi: 10.1002/etc.5530 (PMC10107632; doi:10.1002/etc.5530)
Supplement: Supplementary file 1 — Supplementary information. [file ETC-42-303-s001.docx]

**Improving the Measurement of Iron(III) Bioavailability in Freshwater Samples – Methods and Performance**

**SUPPLEMENTARY TABLES AND FIGURES**

**MATERIALS AND METHODS**

Table S1. Details of the coordinates for each sampling site in The Pilbara region, Western Australia.

| **Sample ID** | **Coordinates** |
| --- | --- |
| Site 1 | 22°13'03.2"S 117°15'10.3"E |
| Site 2 | 22°38'24.8"S 116°47'01.1"E |
| Site 3 | 22°23'03.3"S 116°36'36.9"E |
| Site 4 | 22°17'35.7"S 116°34'23.0"E |

**RESULTS**

Figure S1. XRD spectrum showing the matching of iron oxide and oxyhydroxide peaks of the synthetic iron mineral mix in comparison to the filed samples collected in The Pilbara region, Western Australia. Tags show hematite (Hm), goethite (Gt), magnetite (Mt), and quartz (Qz) peak location (degrees 2θ).


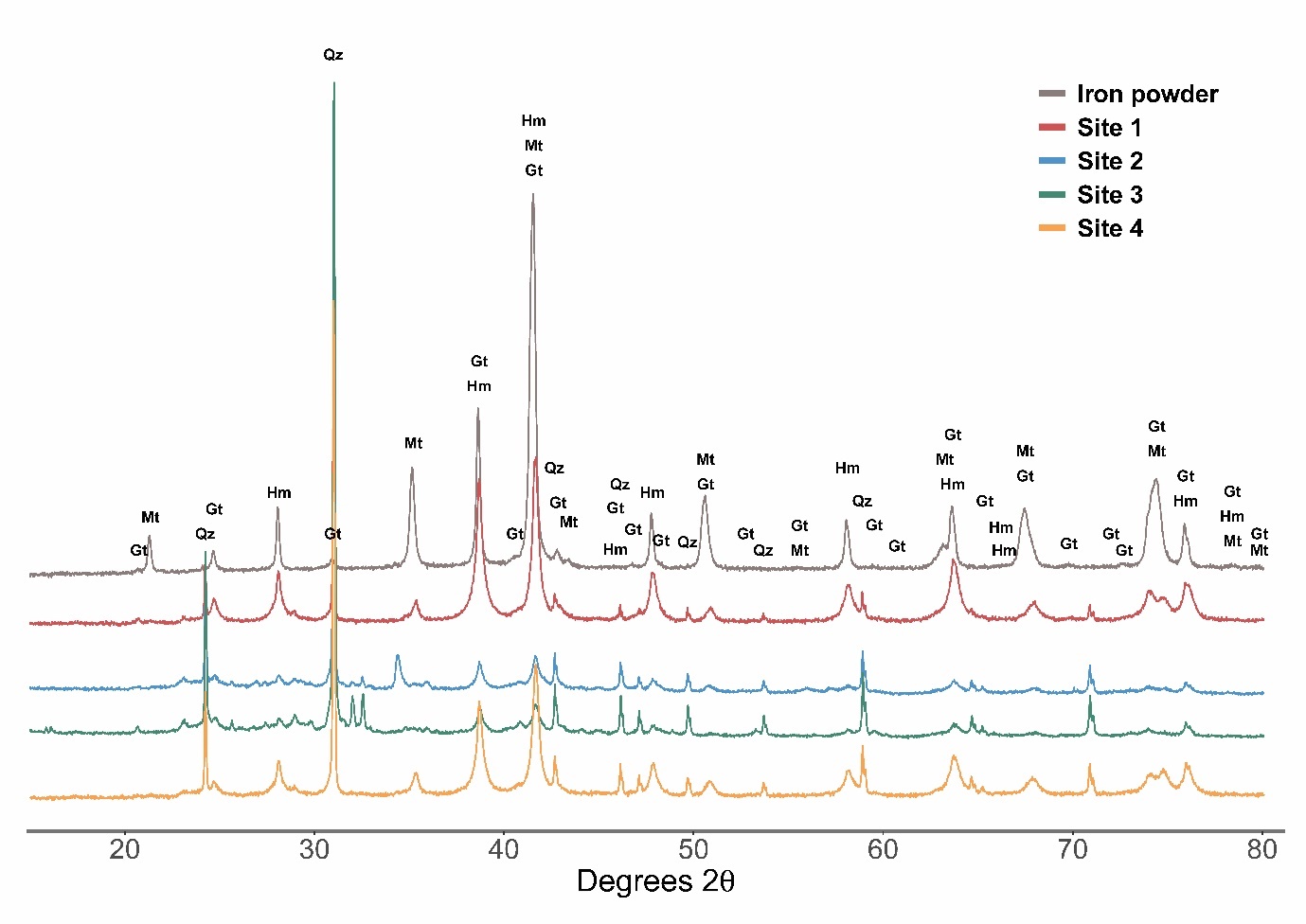


Table S2. Mean iron concentration and standard deviation (SD) in the blanks of the pH 2 experiments. Mean values were calculated from triplicates.

| **Fraction** | **Treatment** | **Age (days)** | **Time (hours)** | **Fe (mg/L)** | **SD (mg/L)** |
| --- | --- | --- | --- | --- | --- |
| Filtered | Blank | 1 | 0 | 0.001 | 0.001 |
| Acidified pH 2 | Blank | 1 | 0.5 | 0.000 | 0.001 |
| Acidified pH 2 | Blank | 1 | 2 | 0.003 | 0.002 |
| Acidified pH 2 | Blank | 1 | 4 | 0.003 | 0.002 |
| Acidified pH 2 | Blank | 1 | 8 | 0.003 | 0.002 |
| Acidified pH 2 | Blank | 1 | 16 | 0.002 | 0.001 |
| Filtered | Blank | 3 | 0 | 0.000 | 0.001 |
| Acidified pH 2 | Blank | 3 | 0.5 | 0.000 | 0.001 |
| Acidified pH 2 | Blank | 3 | 2 | 0.002 | 0.003 |
| Acidified pH 2 | Blank | 3 | 4 | 0.001 | 0.001 |
| Acidified pH 2 | Blank | 3 | 8 | 0.003 | 0.002 |
| Acidified pH 2 | Blank | 3 | 16 | 0.003 | 0.003 |
| Filtered | Blank | 7 | 0 | 0.001 | 0.003 |
| Acidified pH 2 | Blank | 7 | 0.5 | 0.000 | 0.001 |
| Acidified pH 2 | Blank | 7 | 2 | 0.004 | 0.003 |
| Acidified pH 2 | Blank | 7 | 4 | 0.003 | 0.002 |
| Acidified pH 2 | Blank | 7 | 8 | 0.002 | 0.002 |
| Acidified pH 2 | Blank | 7 | 16 | 0.002 | 0.002 |
| Filtered | Blank | 14 | 0 | 0.003 | 0.001 |
| Acidified pH 2 | Blank | 14 | 0.5 | 0.000 | 0.001 |
| Acidified pH 2 | Blank | 14 | 2 | 0.001 | 0.001 |
| Acidified pH 2 | Blank | 14 | 4 | 0.001 | 0.008 |
| Acidified pH 2 | Blank | 14 | 8 | 0.001 | 0.002 |
| Acidified pH 2 | Blank | 14 | 16 | 0.003 | 0.002 |
| Total Recoverable | Blank |  |  | 0.05 | 0.03 |

Table S3. Mean iron concentration and standard deviation (SD) of the single- and combined-phase treatments in the pH 2 experiments. Mean values were calculated from triplicates.

| **Fraction** | **Treatment** | **Age (days)** | **Time (hours)** | **Fe (mg/L)** | **SD (mg/L)** |
| --- | --- | --- | --- | --- | --- |
| Acidified pH 2 | Spiked | 1 | 0.5 | 1.00 | 0.04 |
| Acidified pH 2 | Spiked | 1 | 2 | 1.07 | 0.05 |
| Acidified pH 2 | Spiked | 1 | 4 | 1.09 | 0.04 |
| Acidified pH 2 | Spiked | 1 | 8 | 1.11 | 0.05 |
| Acidified pH 2 | Spiked | 1 | 16 | 1.09 | 0.05 |
| Acidified pH 2 | Spiked | 3 | 0.5 | 0.79 | 0.03 |
| Acidified pH 2 | Spiked | 3 | 2 | 1.02 | 0.05 |
| Acidified pH 2 | Spiked | 3 | 4 | 1.13 | 0.05 |
| Acidified pH 2 | Spiked | 3 | 8 | 1.18 | 0.06 |
| Acidified pH 2 | Spiked | 3 | 16 | 1.16 | 0.04 |
| Acidified pH 2 | Spiked | 7 | 0.5 | 0.70 | 0.02 |
| Acidified pH 2 | Spiked | 7 | 2 | 1.06 | 0.03 |
| Acidified pH 2 | Spiked | 7 | 4 | 1.12 | 0.04 |
| Acidified pH 2 | Spiked | 7 | 8 | 1.14 | 0.06 |
| Acidified pH 2 | Spiked | 7 | 16 | 1.15 | 0.04 |
| Acidified pH 2 | Spiked | 14 | 0.5 | 0.52 | 0.03 |
| Acidified pH 2 | Spiked | 14 | 2 | 0.91 | 0.03 |
| Acidified pH 2 | Spiked | 14 | 4 | 1.04 | 0.04 |
| Acidified pH 2 | Spiked | 14 | 8 | 1.10 | 0.04 |
| Acidified pH 2 | Spiked | 14 | 16 | 1.13 | 0.03 |
| Total | Spiked |  |  | 1.20 | 0.09 |
| Acidified pH 2 | Particulate | 1 | 0.5 | 0.09 | 0.01 |
| Acidified pH 2 | Particulate | 1 | 2 | 0.12 | 0.01 |
| Acidified pH 2 | Particulate | 1 | 4 | 0.14 | 0.01 |
| Acidified pH 2 | Particulate | 1 | 8 | 0.17 | 0.01 |
| Acidified pH 2 | Particulate | 1 | 16 | 0.20 | 0.01 |
| Acidified pH 2 | Particulate | 3 | 0.5 | 0.08 | 0.001 |
| Acidified pH 2 | Particulate | 3 | 2 | 0.11 | 0.004 |
| Acidified pH 2 | Particulate | 3 | 4 | 0.15 | 0.002 |
| Acidified pH 2 | Particulate | 3 | 8 | 0.18 | 0.08 |
| Acidified pH 2 | Particulate | 3 | 16 | 0.22 | 0.01 |
| Acidified pH 2 | Particulate | 7 | 0.5 | 0.07 | 0.003 |
| Acidified pH 2 | Particulate | 7 | 2 | 0.12 | 0.004 |
| Acidified pH 2 | Particulate | 7 | 4 | 0.14 | 0.003 |
| Acidified pH 2 | Particulate | 7 | 8 | 0.17 | 0.01 |
| Acidified pH 2 | Particulate | 7 | 16 | 0.20 | 0.004 |
| Acidified pH 2 | Particulate | 14 | 0.5 | 0.07 | 0.002 |
| Acidified pH 2 | Particulate | 14 | 2 | 0.11 | 0.004 |
| Acidified pH 2 | Particulate | 14 | 4 | 0.16 | 0.03 |
| Acidified pH 2 | Particulate | 14 | 8 | 0.17 | 0.01 |
| Acidified pH 2 | Particulate | 14 | 16 | 0.21 | 0.01 |
| Total | Particulate |  |  | 28.1 | 1.9 |
| Acidified pH 2 | Particulate+Spike | 1 | 0.5 | 1.05 | 0.02 |
| Acidified pH 2 | Particulate+Spike | 1 | 2 | 1.18 | 0.02 |
| Acidified pH 2 | Particulate+Spike | 1 | 4 | 1.20 | 0.02 |
| Acidified pH 2 | Particulate+Spike | 1 | 8 | 1.23 | 0.01 |
| Acidified pH 2 | Particulate+Spike | 1 | 16 | 1.27 | 0.02 |
| Acidified pH 2 | Particulate+Spike | 3 | 0.5 | 0.84 | 0.02 |
| Acidified pH 2 | Particulate+Spike | 3 | 2 | 1.07 | 0.02 |
| Acidified pH 2 | Particulate+Spike | 3 | 4 | 1.20 | 0.01 |
| Acidified pH 2 | Particulate+Spike | 3 | 8 | 1.24 | 0.01 |
| Acidified pH 2 | Particulate+Spike | 3 | 16 | 1.28 | 0.01 |
| Acidified pH 2 | Particulate+Spike | 7 | 0.5 | 0.75 | 0.02 |
| Acidified pH 2 | Particulate+Spike | 7 | 2 | 1.10 | 0.03 |
| Acidified pH 2 | Particulate+Spike | 7 | 4 | 1.19 | 0.06 |
| Acidified pH 2 | Particulate+Spike | 7 | 8 | 1.18 | 0.02 |
| Acidified pH 2 | Particulate+Spike | 7 | 16 | 1.22 | 0.02 |
| Acidified pH 2 | Particulate+Spike | 14 | 0.5 | 0.57 | 0.03 |
| Acidified pH 2 | Particulate+Spike | 14 | 2 | 0.94 | 0.01 |
| Acidified pH 2 | Particulate+Spike | 14 | 4 | 1.04 | 0.02 |
| Acidified pH 2 | Particulate+Spike | 14 | 8 | 1.13 | 0.02 |
| Acidified pH 2 | Particulate+Spike | 14 | 16 | 1.19 | 0.02 |
| Total | Particulate+Spike |  |  | 32.0 | 1.03 |

Table S4. Mean iron concentration and standard deviation (SD) in the blanks of the pH 4 experiments. Mean values were calculated from triplicates.

| **Fraction** | **Treatment** | **Age (days)** | **Time (hours)** | **Fe (mg/L)** | **SD (mg/L)** |
| --- | --- | --- | --- | --- | --- |
| Filtered | Blank | 1 | 0 | 0.006 | 0.00 |
| Acidified pH 4 | Blank | 1 | 0.5 | 0.006 | 0.00 |
| Acidified pH 4 | Blank | 1 | 2 | 0.004 | 0.00 |
| Acidified pH 4 | Blank | 1 | 4 | 0.004 | 0.00 |
| Acidified pH 4 | Blank | 1 | 8 | 0.006 | 0.00 |
| Acidified pH 4 | Blank | 1 | 16 | 0.005 | 0.00 |
| Filtered | Blank | 3 | 0 | 0.006 | 0.00 |
| Acidified pH 4 | Blank | 3 | 0.5 | 0.005 | 0.00 |
| Acidified pH 4 | Blank | 3 | 2 | 0.004 | 0.00 |
| Acidified pH 4 | Blank | 3 | 4 | 0.005 | 0.00 |
| Acidified pH 4 | Blank | 3 | 8 | 0.003 | 0.00 |
| Acidified pH 4 | Blank | 3 | 16 | 0.003 | 0.00 |
| Filtered | Blank | 7 | 0 | 0.007 | 0.00 |
| Acidified pH 4 | Blank | 7 | 0.5 | 0.005 | 0.00 |
| Acidified pH 4 | Blank | 7 | 2 | 0.006 | 0.00 |
| Acidified pH 4 | Blank | 7 | 4 | 0.006 | 0.00 |
| Acidified pH 4 | Blank | 7 | 8 | 0.002 | 0.00 |
| Acidified pH 4 | Blank | 7 | 16 | 0.006 | 0.00 |
| Filtered | Blank | 14 | 0 | 0.007 | 0.00 |
| Acidified pH 4 | Blank | 14 | 0.5 | 0.003 | 0.00 |
| Acidified pH 4 | Blank | 14 | 2 | 0.003 | 0.00 |
| Acidified pH 4 | Blank | 14 | 4 | 0.004 | 0.00 |
| Acidified pH 4 | Blank | 14 | 8 | 0.006 | 0.00 |
| Acidified pH 4 | Blank | 14 | 16 | 0.005 | 0.00 |
| Total | Blank |  |  | 0.021 | 0.002 |

Table S5. Mean iron concentration and standard deviation (SD) of the single- and combined-phase treatments in the pH 4 experiments. Mean values were calculated from triplicates.

| **Fraction** | **Treatment** | **Age (days)** | **Time (hours)** | **Fe (mg/L)** | | **SD (mg/L)** |
| --- | --- | --- | --- | --- | --- | --- |
| Acidified pH 4 | Spiked | 1 | 0.5 | 0.16 | | 0.01 |
| Acidified pH 4 | Spiked | 1 | 2 | 0.18 | | 0.02 |
| Acidified pH 4 | Spiked | 1 | 4 | 0.17 | | 0.01 |
| Acidified pH 4 | Spiked | 1 | 8 | 0.20 | | 0.01 |
| Acidified pH 4 | Spiked | 1 | 16 | 0.23 | | 0.03 |
| Acidified pH 4 | Spiked | 3 | 0 | 0.44 | | 0.11 |
| Acidified pH 4 | Spiked | 3 | 0.5 | 0.14 | | 0.01 |
| Acidified pH 4 | Spiked | 3 | 2 | 0.17 | | 0.02 |
| Acidified pH 4 | Spiked | 3 | 4 | 0.18 | | 0.01 |
| Acidified pH 4 | Spiked | 3 | 8 | 0.19 | | 0.02 |
| Acidified pH 4 | Spiked | 3 | 16 | 0.21 | | 0.001 |
| Acidified pH 4 | Spiked | 7 | 0 | 0.30 | | 0.20 |
| Acidified pH 4 | Spiked | 7 | 0.5 | 0.14 | | 0.02 |
| Acidified pH 4 | Spiked | 7 | 2 | 0.18 | | 0.02 |
| Acidified pH 4 | Spiked | 7 | 4 | 0.20 | | 0.02 |
| Acidified pH 4 | Spiked | 7 | 8 | 0.19 | | 0.02 |
| Acidified pH 4 | Spiked | 7 | 16 | 0.21 | | 0.03 |
| Acidified pH 4 | Spiked | 14 | 0 | 0.38 | | 0.04 |
| Acidified pH 4 | Spiked | 14 | 0.5 | 0.14 | | 0.02 |
| Acidified pH 4 | Spiked | 14 | 2 | 0.18 | | 0.03 |
| Acidified pH 4 | Spiked | 14 | 4 | 0.18 | | 0.01 |
| Acidified pH 4 | Spiked | 14 | 8 | 0.22 | | 0.01 |
| Acidified pH 4 | Spiked | 14 | 16 | 0.20 | | 0.01 |
| Total | Spiked |  |  | 1.17 | | 0.03 |
| Acidified pH 4 | Particulate | 1 | 0.5 | | 0.02 | 0.00 |
| Acidified pH 4 | Particulate | 1 | 2 | | 0.02 | 0.00 |
| Acidified pH 4 | Particulate | 1 | 4 | | 0.03 | 0.01 |
| Acidified pH 4 | Particulate | 1 | 8 | | 0.04 | 0.01 |
| Acidified pH 4 | Particulate | 1 | 16 | | 0.05 | 0.02 |
| Acidified pH 4 | Particulate | 3 | 0 | | 0.01 | 0.003 |
| Acidified pH 4 | Particulate | 3 | 0.5 | | 0.02 | 0.00 |
| Acidified pH 4 | Particulate | 3 | 2 | | 0.02 | 0.00 |
| Acidified pH 4 | Particulate | 3 | 4 | | 0.02 | 0.00 |
| Acidified pH 4 | Particulate | 3 | 8 | | 0.03 | 0.00 |
| Acidified pH 4 | Particulate | 3 | 16 | | 0.04 | 0.00 |
| Acidified pH 4 | Particulate | 7 | 0 | | 0.01 | 0.00 |
| Acidified pH 4 | Particulate | 7 | 0.5 | | 0.02 | 0.00 |
| Acidified pH 4 | Particulate | 7 | 2 | | 0.02 | 0.00 |
| Acidified pH 4 | Particulate | 7 | 4 | | 0.03 | 0.00 |
| Acidified pH 4 | Particulate | 7 | 8 | | 0.03 | 0.00 |
| Acidified pH 4 | Particulate | 7 | 16 | | 0.03 | 0.00 |
| Acidified pH 4 | Particulate | 14 | 0 | | 0.01 | 0.01 |
| Acidified pH 4 | Particulate | 14 | 0.5 | | 0.01 | 0.00 |
| Acidified pH 4 | Particulate | 14 | 2 | | 0.02 | 0.00 |
| Acidified pH 4 | Particulate | 14 | 4 | | 0.03 | 0.00 |
| Acidified pH 4 | Particulate | 14 | 8 | | 0.04 | 0.01 |
| Acidified pH 4 | Particulate | 14 | 16 | | 0.04 | 0.00 |
| Total | Particulate |  |  | | 31.9 | 2.79 |
| Acidified pH 4 | Spiked + Particulate | 1 | 0.5 | | 0.26 | 0.07 |
| Acidified pH 4 | Spiked + Particulate | 1 | 2 | | 0.23 | 0.06 |
| Acidified pH 4 | Spiked + Particulate | 1 | 4 | | 0.25 | 0.06 |
| Acidified pH 4 | Spiked + Particulate | 1 | 8 | | 0.22 | 0.03 |
| Acidified pH 4 | Spiked + Particulate | 1 | 16 | | 0.23 | 0.03 |
| Acidified pH 4 | Spiked + Particulate | 3 | 0 | | 0.64 | 0.15 |
| Acidified pH 4 | Spiked + Particulate | 3 | 0.5 | | 0.22 | 0.07 |
| Acidified pH 4 | Spiked + Particulate | 3 | 2 | | 0.23 | 0.07 |
| Acidified pH 4 | Spiked + Particulate | 3 | 4 | | 0.22 | 0.06 |
| Acidified pH 4 | Spiked + Particulate | 3 | 8 | | 0.20 | 0.04 |
| Acidified pH 4 | Spiked + Particulate | 3 | 16 | | 0.19 | 0.02 |
| Acidified pH 4 | Spiked + Particulate | 7 | 0 | | 0.57 | 0.23 |
| Acidified pH 4 | Spiked + Particulate | 7 | 0.5 | | 0.23 | 0.12 |
| Acidified pH 4 | Spiked + Particulate | 7 | 2 | | 0.26 | 0.11 |
| Acidified pH 4 | Spiked + Particulate | 7 | 4 | | 0.23 | 0.07 |
| Acidified pH 4 | Spiked + Particulate | 7 | 8 | | 0.20 | 0.08 |
| Acidified pH 4 | Spiked + Particulate | 7 | 16 | | 0.20 | 0.06 |
| Acidified pH 4 | Spiked + Particulate | 14 | 0 | | 0.44 | 0.15 |
| Acidified pH 4 | Spiked + Particulate | 14 | 0.5 | | 0.20 | 0.10 |
| Acidified pH 4 | Spiked + Particulate | 14 | 2 | | 0.25 | 0.09 |
| Acidified pH 4 | Spiked + Particulate | 14 | 4 | | 0.21 | 0.08 |
| Acidified pH 4 | Spiked + Particulate | 14 | 8 | | 0.23 | 0.06 |
| Acidified pH 4 | Spiked + Particulate | 14 | 16 | | 0.20 | 0.06 |
| Total | Spiked + Particulate |  |  | | 36.3 | 1.18 |

Table S6. Mean iron concentration, standard deviation (SD) and recovery for all ageing groups and extraction times 0.5, 4, and 16 hours in the spiked-only treatment in the pH 2 experiments. Recovery was calculated with respect to the total recoverable fraction.

| **Treatment** | **Time (hours)** | **Age (days)** | **Fe**  **(mg /L)** | **SD**  **(mg /L)** | **Recovery (%)** |
| --- | --- | --- | --- | --- | --- |
| Single  Spiked | 16 | 1 | 1.09 | 0.05 | 91.9 |
|  |  | 3 | 1.16 | 0.04 | 96.5 |
|  |  | 7 | 1.15 | 0.04 | 95.8 |
|  |  | 14 | 1.13 | 0.03 | 93.7 |
|  | 4 | 1 | 1.09 | 0.04 | 91.1 |
|  |  | 3 | 1.13 | 0.05 | 94.5 |
|  |  | 7 | 1.12 | 0.04 | 93.2 |
|  |  | 14 | 1.04 | 0.04 | 86.7 |
|  | 0.5 | 1 | 1.00 | 0.04 | 83.0 |
|  |  | 3 | 0.79 | 0.03 | 65.5 |
|  |  | 7 | 0.70 | 0.02 | 58.0 |
|  |  | 14 | 0.52 | 0.03 | 43.0 |

Table S7. One-way ANOVA results comparing recovery among age groups for each treatment of the pH 2 extraction.

| **Treatment** | **Extraction time pH 2 (hours)** | **F value** | **p value** |
| --- | --- | --- | --- |
| Single  Spiked | 0.5 | 131 | < 0.001 |
|  | 2 | 9.25 | 5.59 x10^-3^ |
|  | 4 | 2.96 | 0.10 |
|  | 8 | 1.30 | 0.34 |
|  | 16 | 1.74 | 0.24 |
| Single  Particulate | 0.5 | 20.0 | < 0.001 |
|  | 2 | 2.40 | 0.14 |
|  | 4 | 0.78 | 0.54 |
|  | 8 | 1.26 | 0.35 |
|  | 16 | 1.73 | 0.24 |
| Combined | 0.5 h | 187 | < 0.001 |
|  | 2 | 78.8 | < 0.001 |
|  | 4 | 14.2 | 1.44 x10^-3^ |
|  | d 8 | 23.4 | < 0.001 |
|  | 16 | 16.1 | < 0.001 |

Table S8. Mean iron concentration, standard deviation (SD) and recovery for all ageing groups and extraction times 0.5, 4, and 16 hours in the particulate-only treatment with mineralised iron in the pH 2 experiments. Recovery was calculated with respect to the total recoverable fraction.

| **Treatment** | **Time (hours)** | **Age (days)** | **Fe**  **(mg /L)** | **SD**  **(mg /L)** | **Recovery (%)** |
| --- | --- | --- | --- | --- | --- |
| Single  Particulate | 16 | 1 | 0.20 | 0.01 | 0.73 |
|  |  | 3 | 0.22 | 0.01 | 0.76 |
|  |  | 7 | 0.20 | 0.004 | 0.72 |
|  |  | 14 | 0.21 | 0.01 | 0.76 |
|  | 4 | 1 | 0.14 | 0.01 | 0.50 |
|  |  | 3 | 0.15 | 0.002 | 0.53 |
|  |  | 7 | 0.14 | 0.003 | 0.50 |
|  |  | 14 | 0.16 | 0.03 | 0.57 |
|  | 0.5 | 1 | 0.09 | 0.01 | 0.32 |
|  |  | 3 | 0.08 | 0.001 | 0.27 |
|  |  | 7 | 0.07 | 0.003 | 0.26 |
|  |  | 14 | 0.07 | 0.002 | 0.24 |

Table S9. Mean iron concentration, standard deviation (SD) and recovery for all ageing groups and extraction times 0.5, 4, and 16 hours in the combined treatment in the pH 2 experiments. Recovery was calculated with respect to the total recoverable fraction.

| **Treatment** | **Time (hours)** | **Age (days)** | **Fe (mg /L)** | **SD (mg /L)** | **Recovery (%)** |
| --- | --- | --- | --- | --- | --- |
| Combined | 16 | 1 | 1.26 | 0.02 | 3.92 |
|  |  | 3 | 1.28 | 0.01 | 4.00 |
|  |  | 7 | 1.22 | 0.02 | 3.81 |
|  |  | 14 | 1.19 | 0.02 | 3.72 |
|  | 4 | 1 | 1.20 | 0.02 | 3.75 |
|  |  | 3 | 1.20 | 0.01 | 3.74 |
|  |  | 7 | 1.19 | 0.06 | 3.73 |
|  |  | 14 | 1.04 | 0.02 | 3.26 |
|  | 0.5 | 1 | 1.05 | 0.02 | 3.29 |
|  |  | 3 | 0.84 | 0.02 | 2.63 |
|  |  | 7 | 0.75 | 0.02 | 2.34 |
|  |  | 14 | 0.57 | 0.03 | 1.77 |

Table S10. Mean iron concentration, standard deviation (SD) and recovery for all ageing groups and extraction times 0.5, 4, and 16 hours in the spiked-only treatment in the pH 4 experiments. Recovery was calculated with respect to the total recoverable fraction.

| **Treatment** | **Time (hours)** | **Age (days)** | **Fe (mg /L)** | **SD (mg /L)** | **Recovery (%)** |
| --- | --- | --- | --- | --- | --- |
| Single  Spiked | 16 | 1 | 0.23 | 0.03 | 19.2 |
|  |  | 3 | 0.21 | 0.00 | 18.3 |
|  |  | 7 | 0.21 | 0.03 | 17.8 |
|  |  | 14 | 0.19 | 0.01 | 16.6 |
|  | 4 | 1 | 0.17 | 0.01 | 14.8 |
|  |  | 3 | 0.18 | 0.01 | 15.7 |
|  |  | 7 | 0.20 | 0.02 | 17.4 |
|  |  | 14 | 0.18 | 0.01 | 15.5 |
|  | 0.5 | 1 | 0.16 | 0.01 | 13.8 |
|  |  | 3 | 0.14 | 0.01 | 11.9 |
|  |  | 7 | 0.14 | 0.02 | 11.6 |
|  |  | 14 | 0.14 | 0.02 | 11.7 |

Table S11. Mean iron concentration, standard deviation (SD) and recovery for all ageing groups and extraction times 0.5, 4, and 16 hours in the particulate-only treatment in the pH 4 experiments. Recovery was calculated with respect to the total recoverable fraction.

| **Treatment** | **Time (hours)** | **Age (days)** | **Fe**  **(mg /L)** | **SD**  **(mg /L)** | **Recovery (%)** |
| --- | --- | --- | --- | --- | --- |
| Single  Particulate | 16 | 1 | 0.05 | 0.02 | 0.17 |
|  |  | 3 | 0.04 | 0.003 | 0.12 |
|  |  | 7 | 0.03 | 0.001 | 0.11 |
|  |  | 14 | 0.04 | 0.001 | 0.11 |
|  | 4 | 1 | 0.03 | 0.01 | 0.10 |
|  |  | 3 | 0.02 | 0.001 | 0.08 |
|  |  | 7 | 0.03 | 0.003 | 0.09 |
|  |  | 14 | 0.03 | 0.003 | 0.08 |
|  | 0.5 | 1 | 0.02 | 0.001 | 0.05 |
|  |  | 3 | 0.02 | 0.01 | 0.06 |
|  |  | 7 | 0.02 | 0.004 | 0.05 |
|  |  | 14 | 0.01 | 0.003 | 0.04 |

Table S12. Mean iron concentration, standard deviation (SD) and recovery for all ageing groups and extraction times 0.5, 4, and 16 hours in the combined treatment in the pH 4 experiments. Recovery was calculated with respect to the total recoverable fraction.

| **Treatment** | **Time (hours)** | **Age (days)** | **Fe (mg /L)** | **SD (mg /L)** | **Recovery (%)** |
| --- | --- | --- | --- | --- | --- |
| Combined | 16 | 1 | 0.23 | 0.03 | 0.64 |
|  |  | 3 | 0.19 | 0.02 | 0.53 |
|  |  | 7 | 0.20 | 0.06 | 0.54 |
|  |  | 14 | 0.20 | 0.06 | 0.54 |
|  | 4 | 1 | 0.25 | 0.06 | 0.69 |
|  |  | 3 | 0.22 | 0.06 | 0.60 |
|  |  | 7 | 0.23 | 0.07 | 0.64 |
|  |  | 14 | 0.21 | 0.08 | 0.59 |
|  | 0.5 | 1 | 0.26 | 0.07 | 0.71 |
|  |  | 3 | 0.22 | 0.07 | 0.62 |
|  |  | 7 | 0.23 | 0.12 | 0.63 |
|  |  | 14 | 0.20 | 0.10 | 0.56 |

Table S13. One-way ANOVA results comparing recovery among age groups for each treatment of the pH 4 extraction.

| **Treatment** | **Extraction time pH 4 (hours)** | **F value** | **p value** |
| --- | --- | --- | --- |
| Single  Spiked | 0.5 | 1.44 | 0.30 |
|  | 2 | 0.18 | 0.90 |
|  | 4 | 2.58 | 0.13 |
|  | 8 | 1.12 | 0.40 |
|  | 16 | 1.12 | 0.40 |
| Single  Particulate | 0.5 | 2.11 | 0.18 |
|  | 2 | 1.78 | 0.23 |
|  | 4 | 2.39 | 0.14 |
|  | 8 | 0.17 | 0.91 |
|  | 16 | 0.17 | 0.91 |
| Combined | 0.5 | 0.18 | 0.91 |
|  | 2 | 0.10 | 0.96 |
|  | 4 | 0.17 | 0.92 |
|  | d 8 | 0.67 | 0.59 |
|  | 16 | 0.67 | 0.59 |


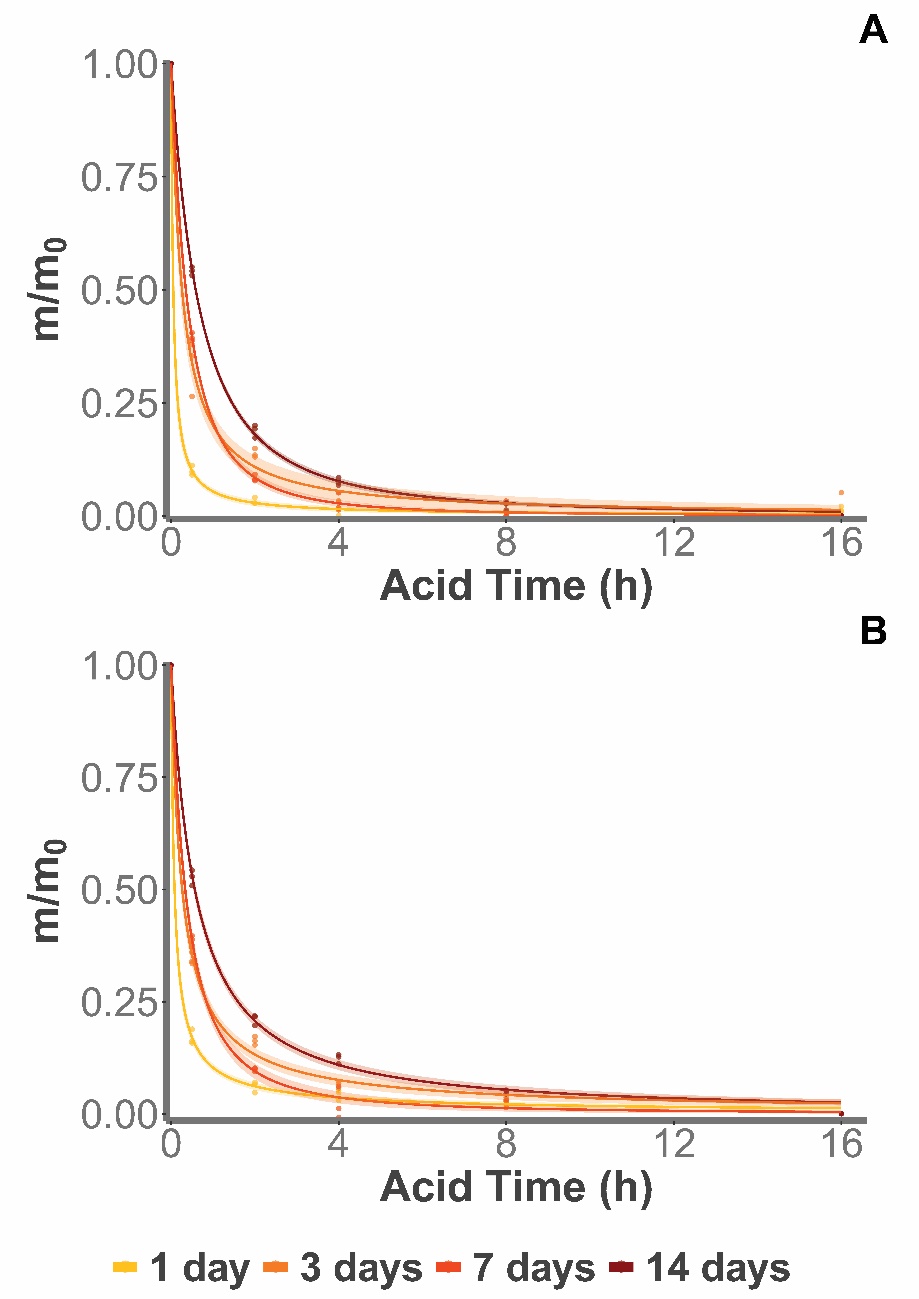
Figure S2. Model fits of the remaining solid fraction (m/m_0_) as a function of pH 2 extraction time (hours) for each ageing group in the spiked-only (A) and combined (B) treatments. Shading indicates 95% confidence intervals.

Table S14. Cross-sectional weighted linear regression of the log-transformed initial dissolution rate as a function of ageing time for the spiked-only treatment in the pH 2 experiments.

| **Treatment** | **Coefficients** | **Estimate** | **SE** | **p value** |
| --- | --- | --- | --- | --- |
| Spiked pH 2 | Intercept | -5.80 | 0.60 | < 0.001 |
|  | Age | -0.16 | 0.07 | 0.03 |


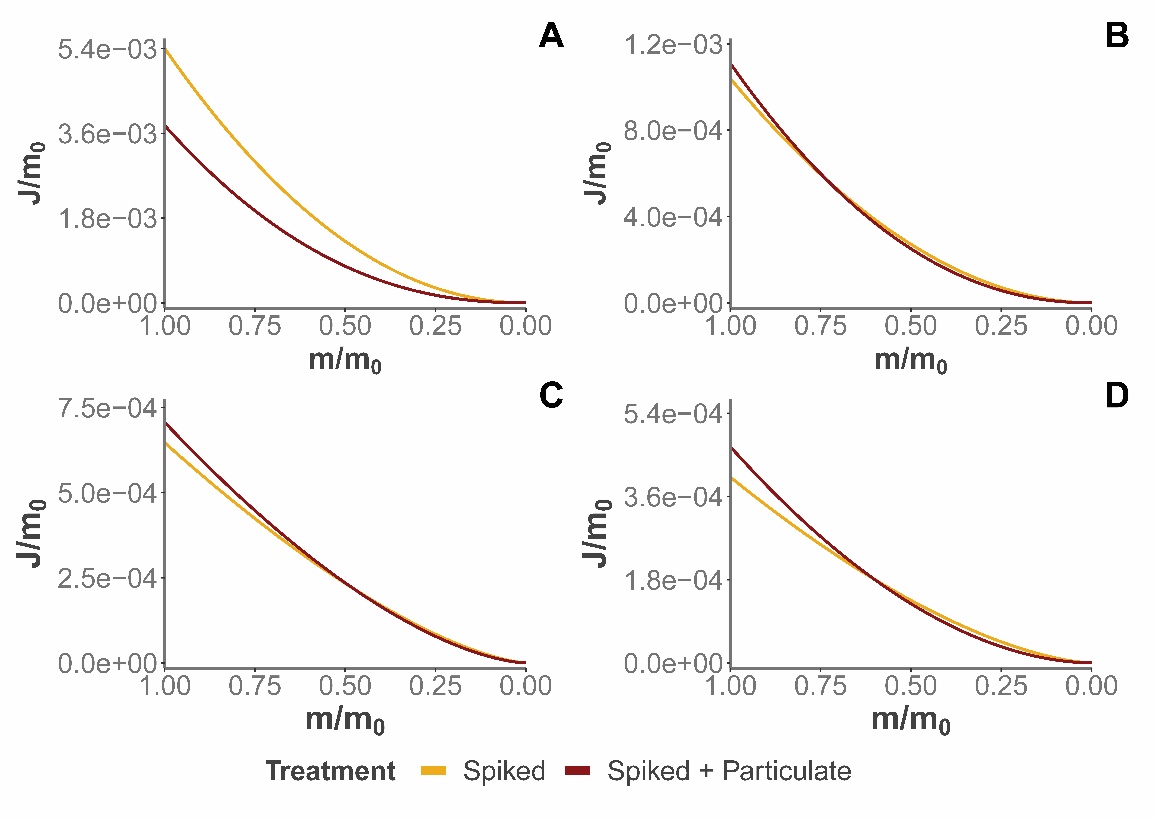
Figure S3. Graphical representation of the overall dissolution rate (J/m_0_) as a function of the remaining solid phase (m/m_0_) for 1-day (A), 3-day (B), 7-day (C), and 14-day (D) old samples for the spiked-only and combined treatments in the pH 2 experiments.

Table S15. Nested model contrasts using the pooled data of the spiked-only and combined treatments. The F-ratio test was used for contrasting separate parameters vs. shared parameter models for each age group in the pH 2 experiments.

| **Age (days)** | **F value** | **p value** |
| --- | --- | --- |
| 1 | 54.5 | < 0.001 |
| 3 | 1.86 | 0.17 |
| 7 | 0.89 | 0.42 |
| 14 | 13.7 | < 0.001 |

Table S16. Cross-sectional weighted linear regression of the log-transformed initial dissolution rate as a function of ageing time for the combined treatment in the pH 2 experiments.

| **Treatment** | **Coefficients** | **Estimate** | **SE** | **p value** |
| --- | --- | --- | --- | --- |
| Combined pH 2 | Intercept | -5.99 | 0.44 | < 0.001 |
|  | Age | -0.14 | 0.055 | 0.014 |


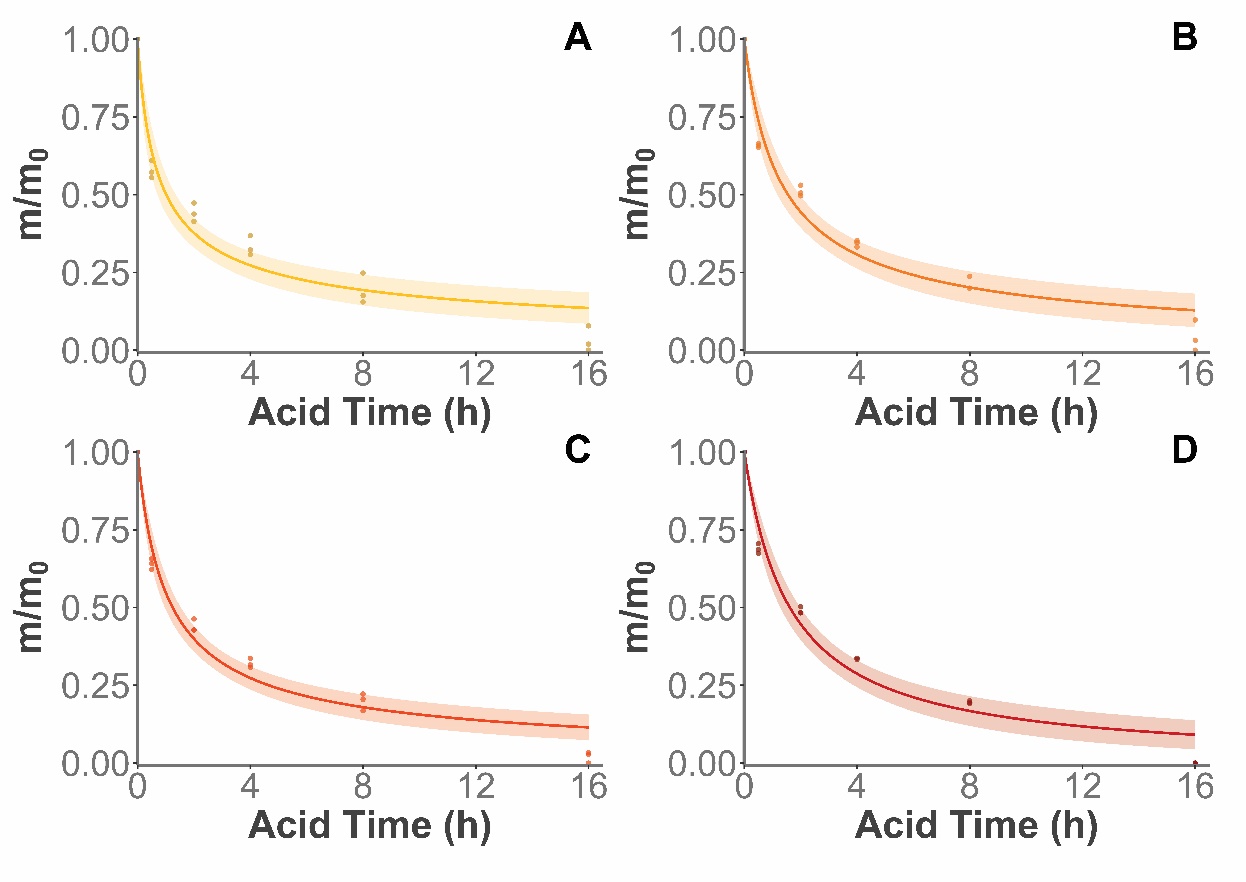
Figure S4. Full-data model fits of the remaining solid fraction (m_t_/m_0_) as a function of pH 2 extraction time (hours) for 1-day (A), 3-day (B), 7-day (C), and 14-day (D) old samples of the particulate-only. Shading indicates 95% confidence intervals.


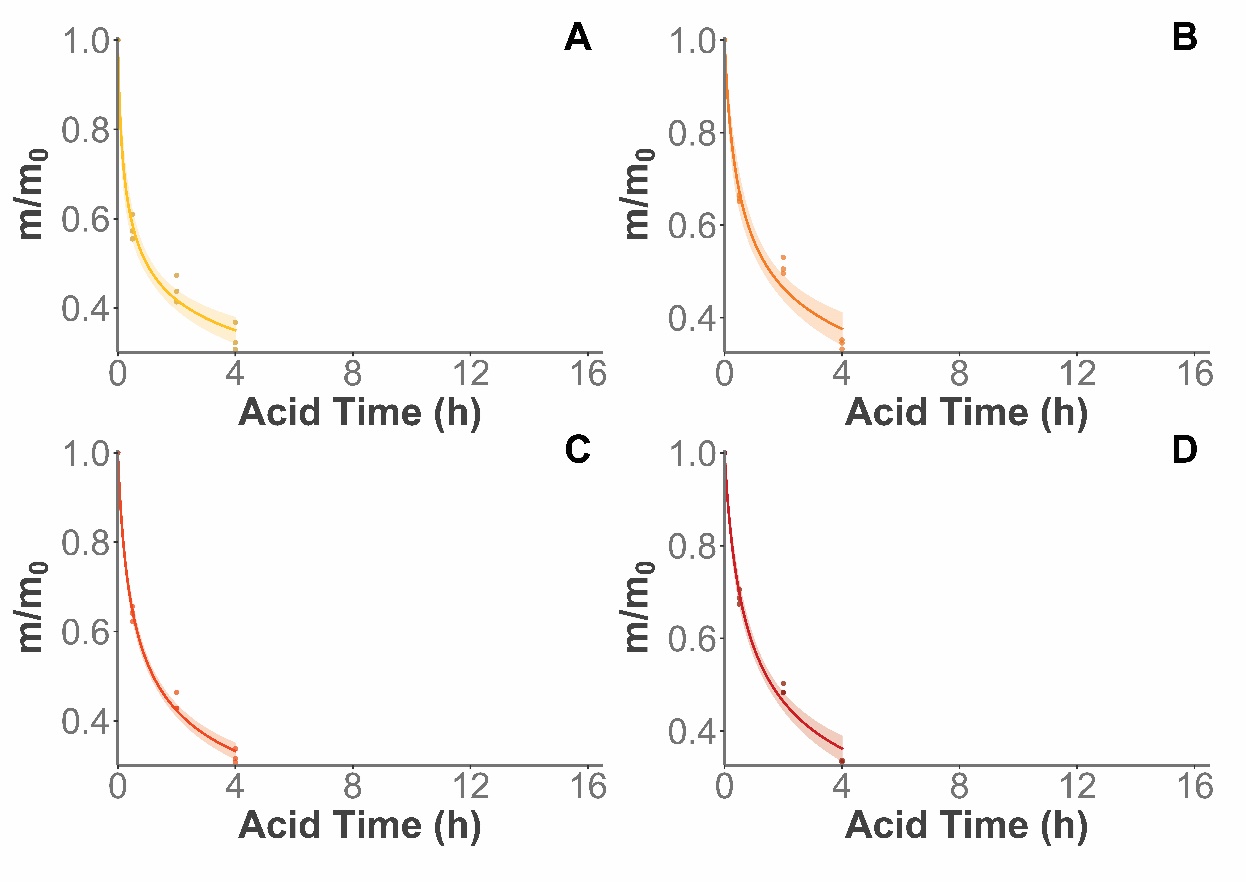
Figure S5. Trimmed-data model fits of the remaining solid fraction (m_t_/m_0_) as a function of pH 2 extraction time (hours) for 1-day (A), 3-day (B), 7-day (C), and 14-day (D) old samples of the particulate-only. Shading indicates 95% confidence intervals.

Table S17. Nested model contrasts of the full-data fits vs. the trimmed-data fits for the single- and combined-phase treatments. The differences detected in the particulate- only treatment showed the trimmed data yielded a better model fitting of the initial dissolution process.

| **Model Contrast** | **Treatment** | **Age (days)** | **F value** | **p value** |
| --- | --- | --- | --- | --- |
| Full data vs. trimmed data | Spiked | 1 | 1.52 | 0.27 |
|  | Spiked | 2 | 0.59 | 0.73 |
|  | Spiked | 3 | 1.04 | 0.46 |
|  | Spiked | 4 | 1.09 | 0.43 |
|  | Spiked + Particulate | 1 | 0.75 | 0.63 |
|  | Spiked + Particulate | 2 | 1.35 | 0.32 |
|  | Spiked + Particulate | 3 | 0.98 | 0.49 |
|  | Spiked + Particulate | 4 | 1.06 | 0.45 |
|  | Particulate | 1 | 15.0 | < 0.001 |
|  | Particulate | 2 | 9.57 | < 0.001 |
|  | Particulate | 3 | 25.1 | < 0.001 |
|  | Particulate | 4 | 16.9 | < 0.001 |
